# Time Scale Hierarchies in the Functional Organization of Complex Behaviors

**DOI:** 10.1371/journal.pcbi.1002198

**Published:** 2011-09-29

**Authors:** Dionysios Perdikis, Raoul Huys, Viktor K. Jirsa

**Affiliations:** Theoretical Neuroscience Group, UMR6233, Institut Science du Mouvement, University of the Mediterranean, Marseille, France; University of California Santa Barbara, United States of America

## Abstract

Traditional approaches to cognitive modelling generally portray cognitive events in terms of ‘discrete’ states (point attractor dynamics) rather than in terms of processes, thereby neglecting the time structure of cognition. In contrast, more recent approaches explicitly address this temporal dimension, but typically provide no entry points into cognitive categorization of events and experiences. With the aim to incorporate both these aspects, we propose a framework for functional architectures. Our approach is grounded in the notion that arbitrary complex (human) behaviour is decomposable into functional modes (elementary units), which we conceptualize as low-dimensional dynamical objects (structured flows on manifolds). The ensemble of modes at an agent’s disposal constitutes his/her functional repertoire. The modes may be subjected to additional dynamics (termed operational signals), in particular, instantaneous inputs, and a mechanism that sequentially selects a mode so that it temporarily dominates the functional dynamics. The inputs and selection mechanisms act on faster and slower time scales then that inherent to the modes, respectively. The dynamics across the three time scales are coupled via feedback, rendering the entire architecture autonomous. We illustrate the functional architecture in the context of serial behaviour, namely cursive handwriting. Subsequently, we investigate the possibility of recovering the contributions of functional modes and operational signals from the output, which appears to be possible only when examining the output phase flow (i.e., not from trajectories in phase space or time).

## Author Summary

In most established approaches to cognitive modelling, cognitive events are treated as ‘discrete’ states, thus passing by the continuous nature of cognitive processes. In contrast, some novel approaches explicitly acknowledge cognition’s temporal structure but provides no entry points into cognitive categorization of events and experiences. We attempt to incorporate both aspects in a new framework, which departs from the established idea that complex (human) behaviour is made up of elementary functional ‘building blocks’, referred to as modes. We model these as mathematical objects that are inherently dynamic (i.e., account for change over time). A mechanism sequentially selects the modes required and binds them together to compose complex behaviours. These modes may be subjected to brief inputs. The ensemble of these three ingredients, which influence one another and operate on different time scales, constitutes a functional architecture. We illustrate the architecture via cursive handwriting simulations, and investigate the possibility of recovering the contributions of the architecture from the written word. This appears possible only when focussing on the dynamic modes.

## Introduction

### The organization of human function

Human (and animal) function is thought to emerge from the embedded dynamics of the organism in its natural and social environment [1], [2]. Its phenomenology thus includes overt behavior (for instance motor behavior) as well as processes internal to the organism. By implication, human function comprises multiple interdependent (i.e., coupled) dynamics operating on a diversity of time scales. The ensemble of dynamics and interactions amongst them constitutes a functional architecture. A common theme in biology and the life sciences is that (‘complex’) function is decomposable into elementary functional units (or building blocks) that can thus be considered as the basic components of functional architectures. Functional units should preserve some of their properties invariant among different utilizations (which identifies them as units). As building blocks, they are brought into meaningful relationships (such as concatenation in time) resulting in longer sequences. Consequently, the resulting complex processes exhibit a meaningful hierarchical structure spanning distinct time scales. For instance, movement (or motor) primitives [3]–[6] and motor programs [7], [8] have been proposed as building blocks of complex and sequential movements. In birdsong notes and syllables (groups of notes) are thought to compose hierarchical sets with a crucial role in their production [9] and perception [10]. ‘Temporal primitives’ have been linked with related lexical items in speech perception [11] and are identified elsewhere [12] as “articulatory gestures that constitute the phonemic elements of both speech generation and perception” and thus “the primitives underlying linguistic communication” (p. 188). Cognitive linguistics [13], [14] supposes that language and cognition are based on so-called conceptual schemas and cognitive mechanisms (as e.g., metaphors and blends [15], [16]) to compose conceptual structures as complex as mathematics [16]. In all these instances, ongoing cognitive (in its broadest sense) function results as elementary units are somehow put into a meaningful relationship.

Two approaches, symbolic computation and connectionism, have dominated cognitive modeling over the last decades. Both define static information representations and focus on operations (‘computation’) for the generation of complex function. Symbolic computation explicitly represents information in terms of organized symbols that are combined via syntactic rules [17]. In connectionist models, parallel computation occurs via patterns of activation distributed across the network's nodes [18]–[20]. Hybrid cognitive architectures combine symbolic representations with connectionist learning algorithms [21]. Common to these approaches is that they define functional units as static patterns or ‘states’ onto which cognitive architectures converge in the process of information processing (in this context; the generation of complex function) [22]. Even when dynamics beyond point attractors is included (as in recurrent neural networks [23]–[25]), it is usually broken down into a succession of discrete states (encoding past context) or transients inside a basin of attraction [25] and treated as such. Identifying functional units as static patterns or states, however, is clearly at odds with one of the cornerstones of biology, namely the process. The notion of a process signifies change; basic functional units should thus contain temporal evolution.

In the approach below we explicitly focus on the dynamical phenomenology of human function instead of on computation. Accordingly, we first propose a general framework in which functional modes—dynamical building blocks into which processes are grounded [26]—capture the geometry of elementary processes' dynamics. Functional modes are brought together into meaningful organizations unfolding in time via a ‘selection’ mechanism that ‘turns processes on and off’. Some functional modes operate autonomously, while others require that quasi-instantaneous ‘kicks’ initiate their functioning. Crucially, so-defined functional architectures embed dynamics that operate on the time scale of the basic functions (adhering to the functional modes) as well as a dynamics whose corresponding time scale is defined over the entire eschewing process (adhering to the selection mechanism). A third characteristic times scale pertains to the (typical) involvement of the brief ‘kicks’. In other words, the architecture comprises a time scale hierarchy. Next, we construct an autonomous functional architecture providing proof of concept and illustrating the approach in the context of handwriting. In this specific realization we implement the time scale hierarchy of the functional architecture through a Winner-Take-All (WTA) competition dynamics [27]–[29] on one level of organization, and a dynamical sequencing mechanism on another. We want to stress, however, that while the outline of the functional architecture is general, the implementation is but one realization of numerous possible ones.

### Functional architectures

The cornerstones of our functional architecture are (i) their constitution as low-dimensional phase-flow governed spatiotemporal patterns (functional modes) describing processes; and (ii) a hierarchical multi time-scale organization allowing for pattern competition of the functional modes. Due to a competition of processes one functional mode dominates during a particular time window.

The notion that human function emerges in terms of low-dimensional spatiotemporal dynamic patterns is key to coordination dynamics [30], [31] and, more generally, synergetics [32]. The latter, a physical theory of self-organized pattern formation, postulates that in the proximity of (pattern) instabilities (brought about by critical values of control parameters) the dynamics is separated into fast and slow variables. The fast variables can be adiabatically eliminated by expressing their dynamics as a function of their slow counterparts, in which case the former are ‘enslaved’ by the latter (the ‘slaving principle’). That is, reduced system descriptions for the collective dynamics (order parameters) can be derived. Low-dimensional order parameters thus provide functional representations of high-dimensional system. Synergetics has been successfully applied to the perception of ambivalent patterns [33], [34] as well as to behavioral coordination [35]. Coordination dynamics models the dynamical phenomenology of the emerging patterns in experimental paradigms of bimanual [36], [37], sensorimotor [38], and social coordination [39], and learning [40] as low-dimensional, nonlinear dynamical systems via a few (usually one or two) order parameters (see [30] for an overview).

**Figure 1:**
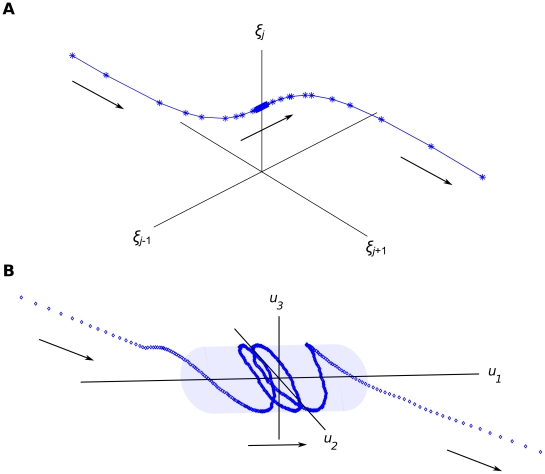
Multiscale dynamics: slow operational signal and SFM emergence. Panel A: The slow operational signals {*ξ_j_*} converge through a fast transient to a specific *ξ_j_* node resulting (here) in the emergence of a cylindrical manifold. Panel B: The functional dynamics {*u_i_*} collapses fast (also) onto the manifold where it executes a slow spiral flow. The *ξ_j_* node's stability is sustained for the duration of the flow execution. Subsequently, the *ξ_j_* node destabilizes, followed by the related manifold and the dynamics moves away, again through fast transients. The density of data point is inversely proportional to the time scale of the dynamics.

Consistent therewith, we adopt the notion that human function is constituted by meaningfully structured low-dimensional patterns, the ‘Structured Flows on Manifolds’ (SFMs; see Figure 1) [41], [42]. Accordingly, during the engagement in a specific function, the functional dynamics adiabatically collapses from an inherently high-dimensional space onto a functionally relevant subset of the phase (state) space, the manifold. On the manifold, a phase flow is prescribed and a trajectory evolves for the duration of the functional process. SFMs aim at linking the dynamics of large-scale brain networks interacting with bodily and environmental dynamics (high-dimensional systems) to low-dimensional phenomenological descriptions of functional (or behavioral) dynamics. Hence, functional processes are ‘encoded’ in terms of structured phase flows, mathematical (structured) entities that unambiguously and quantitatively describe the evolution of autonomous, deterministic, and time-continuous systems in their phase (state) space (see [43] for an introduction). Phase flows not only encode a system's past and future states (given any initial condition and in the absence of stochastic influences) but also its stability and response to perturbations. The vector field describing a flow establishes causal relationships among the system' states by assigning at each state a vector determining the next state. Furthermore, the phase flow topology uniquely determines a system's qualitative behaviour, i.e., it encodes the invariant features of a dynamical process relative to quantitative variation, thus identifying all functional possibilities within a class in a model-independent manner. Indeed, structured phase flows (on low-dimensional manifolds) satisfy the requirement that the dynamics be meaningfully structured, referred to elsewhere as dynamical constituency [44]. In planar systems (systems of two dimensions), common phase flow topologies include point attractors and limit cycles (commonly used to model discrete and rhythmic functions, respectively) and separatrices, that is, structures that locally divide the phase flow into opposing directions, endowing the system with threshold properties and (potentially) multistability [45]. Such 2-dimensional flows have led to (confirmed) counter-intuitive predictions on false starts [46], the discovery of a discontinuity in Fitts' law [47], and the establishment of a taxonomy of discrete and rhythmic movements [48]. For systems of higher (still relatively low) dimensionality, the dynamic repertoire may contain a large variety of functional modes that are in principle adequate to account for elementary processes.

In summary, phase flows can be viewed as functional units that incorporate the properties of low-dimensionality, class-defining invariance together with within-class variation, executive stability (i.e., performance maintenance in the presence of perturbations), meaningful structure (dynamical constituency), and compositionality (i.e., they can be embedded into a larger functional organization. We will use the term *functional modes* to refer to phase flows incorporating this set of properties, and refer to the ensemble of modes that an actor has to his/her proposal as the dynamical repertoire.

The second feature of the functional architecture, i.e., its multi-time scale character, is founded on the fact that complex processes arise in an organism-environment context that inherently covers multiple scales, as the above mentioned examples suggest. Indeed, multiscale architectures have proven a promising choice to describe behavioral, cognitive, or brain dynamics [49]–[53]. Armed with functional modes as essential building blocks, we propose additional dynamics (called *operational signals*) on time scales slower and faster than that of the modes. The slower process effectively binds functional modes together into sequences. More precisely, a given functional mode emerges via a competition process [27]–[29] to temporally dominate the functional dynamics, after which it destabilizes and gives way to another mode. The transient dynamics between modes can be triggered either by ‘internal’ events (as in pre-constructed sequences) or by ‘external’ ones (such as perceptual events). The modes' temporal attractivity guarantees functional robustness, whereas transitions between modes flexibility for meaningful changes. Further variability in the functional dynamics may potentially arise via additional dynamics operating on times scales faster than (or similar to) that of the modes. Accordingly, human function is organized in multilevel dynamical hierarchies.

In sum, functional architectures combine invariant features (phase flows) with those that vary across distinct instances of a functional mode's appearance in an agent's behavior (via multiscale operational signals involved; e.g., due to different contexts). For instance, syllables are (largely) invariant units but their embedding in words and phrases depends on context. See [26] for a classification of operational signals based on time scale hierarchy, computational evidence for the ‘efficiency’ of composing complex behaviors out of simpler ones, and functional architectures in particular.

### Functional architectures for serial behavior

We formulate a functional architecture for serial processes (see [54] for a classic study and [55] for a review), and exemplify it in a specific implementation for cursive handwriting. Handwriting is a typical human behavior involving parallel functions that are related to processing across multiple levels: the linguistic, semantic or word, graphemic, allographic or letter, and stroke level [56], [57]. Observations of a principal periodicity in normal cursive handwriting of ∼5 Hz [58] and a slower one ∼1 Hz (3–4 characters) [59] supports the presence of multiple time scales in cursive handwriting.

As pointed out above, the architecture sequentially ‘selects’ functional modes via a competition process among the slow operational signals. In our handwriting example, the modes code for specific characters (or parts thereof). For a word to arise (functionality), specific modes (characters) have to dominate the functional dynamics at an appropriate serial order. We model the serial order behavior using a variation of Competitive Queuing models (CQ) [60]–[63], a class of state of the art models for serial behavior (for a recent review including behavioral and brain data, see [55]). CQ is based on parallel representations of learned sequences; at each stage of a given sequence, the participating elements compete for their activation following an order of priority. We opted for a CQ variation as these models allows for competition dynamics, which has been successfully used in the context of handwriting and related kinematical phenomena [64]. The CQ is not a defining ingredient of our architecture, however.

The architecture models the interaction of processes acting on different time scales. A dynamical repertoire accounts for the generation of cursively written characters and a slower competitive dynamics (operating on the word-generation scale) activates the corresponding modes at appropriate times. These mechanisms are feedback coupled from the output trajectories to the slow competition. An additional instantaneous operational signal, which is (sometimes) used for movement initiation by providing a meaningful perturbation, is coupled to the modes' and competition dynamics. Word generation thus emerges autonomously from the multi-time scale, high-dimensional system, which is constructed out of simpler constituent ones.

Below, we present the mathematical formulation of the general functional architecture (Methods), after which we provide proof of concept via simulations of a specific architecture generating a desired cursively written word (Results). Subsequently, we show how our framework can be used to identify functional units (i.e., how to decompose human function into its generating components). As will become evident, the latter is not trivial due to the multi-scale hierarchy generating observable trajectories. By implication, the slow dynamics is an envelope of the functional modes' dynamics, rendering the resulting process non-stationary [65], [66]. The difficulty of the functional decomposition is reflected in attempts to identify programming units in serial behavior [54], cursive handwriting, in particular. Based on word presentation – movement initiation reaction times, movement time scaling relative to the number of hypothesized units (strokes, letters, graphemes, or syllables) and their individual movement times, interletter times, and errors or measures of disfluency, syllables [67]–[69], complex graphemes [70] such as digraphs [71], letters [72], [73], and single or pairs of up and/or down strokes [74] have all been ascribed this role. As we will show, however, the decomposition into functional units based on the system' functional output is likely compromised due to the generating system' multi-scale character.

## Methods

### Functional modes

We first briefly review the formulation of Structured Flows on Manifolds (SFM) [41], [42], [75], [76] which reads
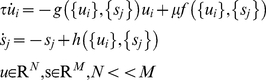
(1)where the so-called ‘smallness’ parameter *µ* is constrained as 0<*µ*<<1, *g*(.) defines the manifold, *f*(.) describes the subsequent flow on it, and *h*(.) represents the fast dynamics that rapidly collapses onto the manifold; here and below *τ* is the time constant of the fast contraction onto the manifold. Due to *µ* being small, the dynamics collapsing on the manifold is much faster than that pertaining to the phase flow. The flow is constrained on the manifold for an appropriate attractive function *g*(.). Unlike the center manifold theory [77], which is a local theory valid around instabilities only, systems of the form of equation (1) need to contain an inertial manifold [78], a global structure used in the reduction of infinite dimensional dynamical systems to finite dimensional spaces. Systems exhibiting inertial manifolds have to be dealt with on a case-by-case basis. SFM can be generated by distributed multi-component systems such as networks of firing-rate neural populations if multiplicative couplings and small connectivity asymmetries are present [41]. The former provide the necessary non-linearities whereas the latter allow for the emergence of the flow on the manifold. Ongoing work [75], [76] attempts to encode SFM into networks of spiking neural populations.

Here, we consider SFM as the macroscopic functional dynamics that emerges from interactions in a high-dimensional system (an agent) under environmental constraints and perturbations. After adiabatically eliminating the fast variables *s_j_* (by solving 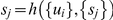 for *s_j_*), the dynamics of a functional mode is described as: 
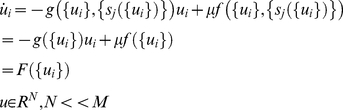
(2)Thus, we consider a functional mode *F*({*u_i_*}) as a (transiently emerging) *N*-dimensional functional dynamics in {*u_i_*} space originating from a ({*u_i_*},{*s_i_*})-space of much higher dimensionality *M*.

### Multi-scale functional architectures

In its most general formulation, we can describe a functional architecture through its flow *F*(.) in phase space potentially subjected to additional operations (for a detailed treatment see [26]):
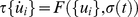
(3)where {*u_i_*} are the system's state variables and *σ*(*t*) is a time-dependent operational signal that, if constant in time (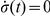), renders the process autonomous. In that case, *F*({*u_i_*}) is identified as the SFM of a particular functional mode. The dynamical repertoire is the set of functional modes available to an agent; it represents the ensemble of elementary functions that appear in relatively invariant manner across different instances of the agent's behavior. In contrast, the operational signals are task-specific dynamics that operate upon the modes in a context-dependent fashion.

Operational signals may evolve on various time scales relative to the functional modes and can in principle span a continuum of scales. Let *τ_f_*  = *τ/µ* and *τ_σ_* denote the time scales corresponding to a particular functional mode and operational signal *σ*(*t*), respectively. Following [26], we distinguish four different instantiations of time scale separations. In cases in which *σ*(*t*) acts much faster than the functional mode (i.e., *τ_σ_*<<*τ_f_*), *σ*(*t*) operates (quasi-instantaneously) upon the mode and we denote it as *δ*(*t*). In cases where *σ*(*t*) acts on a time scale similar to that of the functional mode (i.e., *τ_σ_*≈*τ_f_*), *σ*(*t*) may be said to operate the functional mode, and we write it as *η*(*t*). In cases in which *σ*(*t*) acts much slower than the functional mode (*τ_σ_*>>*τ_f_*), we write it as *ξ*(*t*). Finally, in cases in which *σ*(*t*) can be considered as time-independent (i.e., *σ*(*t*)≈constant during the process; i.e., *τ_σ_*→∞), the mode is autonomous.

Functional modes (*F*({*u_i_*})) and operational signals (*ξ*(*t*), *δ*(*t*)) compose functional architectures in the spirit of physics of pattern formation [28], [32], where spatiotemporal patterns are expressed as a linear combination of a few dominating modes. The critical and novel concept we introduce here is that the modes correspond to elementary processes (expressed as SFM) rather than static spatial patterns. Thus, at each moment in time, the expressed phase flow is given as a linear combination of all functional modes available in an agent's dynamical repertoire:
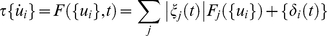
(4)


where {*u_i_*} are the state variables and *F_j_*(.) is the *j*-th mode. *ξ_j_* acts as a weighting coefficient for the *j*-th mode, is constrained to positive values, and operates on a slower time scale than that of the functional modes (even though transitions between modes involving fast contraction on the respective manifold are fast). That is, {*ξ_j_*} ‘select’ a particular mode *F_j_* during its activation phase (when *ξ_j_* = 1 and all other {*ξ_k_*} = 0, for *k≠j*). Figure 1 sketches the resulting multi-scale dynamics as the transient emergence of a spirally structured flow on a cylindrical manifold for the time that the {*ξ_j_*} dynamics stays in the neighborhood of a particular node.

**Figure 2:**
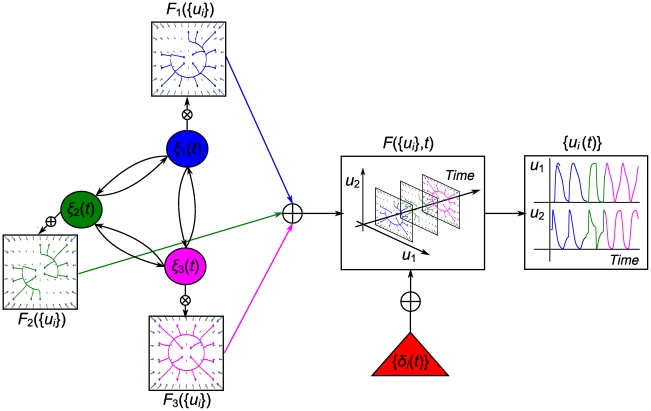
Functional modes and architecture overview. Interactions among functional modes *F_j_*({*u_i_*}) result in one of them dominating the output of the functional architecture for a period of time followed by the domination of another one via a fast transient. Three modes (associated with different colors) of the available dynamic repertoire are shown. They all correspond to 2-dimensional phase flows on an ellipsoid manifold. Blue represents a monostable phase flow, magenta a limit cycle flow, and green a bistable flow. Their vector field and a set of characteristic trajectories starting from different initial conditions (small asterisks) are shown. The modes' mutual interactions are depicted as bidirectional couplings (arrows) among their associated weighting coefficients {ξ_j_(t)} (with which they have a multiplicative relationship). The resulting expressed phase flow F({u_i_},t) (shown as a trajectory in the phase space and time) results from their linear combination at each time moment, while {u_i_(t)} is the respective time series. {ξ_j_(t)} play the role of a slow operating signal (with respect to the inherent time scale of the functional modes, i.e., τ_ξ_>>τ_f_). Finally, an instantaneous (τ_δ_<<τ_f_) operational signal {*δ_i_*(*t*)} (in red) may have an additive contribution to *F*({*u_i_*},*t*), acting like a meaningful perturbation.

Recall, next to the slow dynamics that changes the (expressed) flow topology, the architecture provides for the optional involvement of the instantaneous operational signal {*δ_i_*(*t*)} that does not affect the flow and that acts as a functionally meaningful (context-specific) perturbation. For example, the {*δ_i_*(*t*)} can move the system beyond a threshold (separatrix) and initiate a significant change in the trajectory's evolution. To reiterate, the ensemble of subsystems (*F_j_*({*u_i_*}), *ξ_j_,*{*δ_i_*(*t*)}) operating on distinct time scales (*τ_δ_*<<*τ_f_*<<*τ_ξ_*) constitutes the functional architecture as summarized in Figure 2. In the following sections we provide an illustration of how the {*ξ_j_*} dynamics can be designed to organize functional modes so that more complex functions emerge, in particularly serial order behavior.

### Functional mode competition

We require that the modes' activations do not overlap and implement a ‘Winner-Take-All competition’ (WTA) for the {*ξ_j_*} dynamics: 
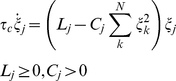
(5)where *τ_c_* is a time constant ensuring that the competition evolves fast, The competition evolves among modes with *L_j_*>0, and its outcome is determined by parameters {*C_j_*} and {*L_j_*}: the ‘winning’ *ξ_j_* is the one with 
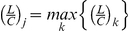
. The competition dynamics has one unstable node at the origin, one point attractor (the ‘winner’) at 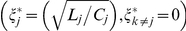, all other equilibrium points being saddles nodes (constraining the dynamics for {*ξ_j_*}>0). (For a linear stability analysis of all equilibrium points of this system, see Text S1; for the phase space of a 2-dimensional WTA competition, see Figure S1 Supporting Information.) Thus, functional modes are organized via mutual competitive interactions. Such a functional mode decomposition based on a competition scheme follows previous work on the Synergetic Computer [28], and is well established in the literature of biological competition [27]. An alternative to the WTA competition could be the winner-less competition based on transient heteroclinic sequences [52], [53], as also used in [75].

### Serial order dynamics

In order to model serial order, suitable dynamics has to be designed so as to activate the appropriate functional modes sequentially with the correct timing. Our here chosen implementation is inspired by Competitive Queuing models of serial behavior [60]–[63] that combine parallel representations of alternative ‘action plans’ with a competition process that selects the action to be executed next. The competition is due to lateral inhibition among the candidate actions; the order of activation depends on a so-called primacy gradient (i.e., a gradient of excitation across the sequence's elements). Every executed action is via inhibitory feedback excluded from the competition for the remaining of the sequence. Accordingly, in our implementation, at each stage of the sequence, the functional modes compete (via the {*ξ_i_*}), one of them wins, dominates the output dynamics for the duration of its activation and is subsequently inhibited, after which the competition continues among the remaining available modes. Equation (5) implements the competition among modes by means of mutual inhibition. The order of activation depends on the {*C_j_*} parameters (the primacy gradient in this case); *L_j_*>0 is the condition for mode *j* to take part at a specific competition round. Thus, parallel representations of the sequence (encoded in the arrays of {*C_j_*} parameters) are combined with serial processes of WTA competition. The timely inhibition of an active mode is achieved through a ‘bottom-up’ coupling (feedback) from the output {*u_i_*} to the slow operational signal: 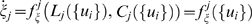.

Here we describe this bottom-up feedback in detail. We introduce the index 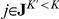, indicating the specific order of the sequence, starting with mode *j* = 1 and terminating at *j* = *K′*, while running among the *K′* modes (out of a repertoire of *K* modes in total) that participate in a sequence **J**. The feedback is mediated by a 2-dimensional differential equation per functional mode of **J** (variables {*ν_j_*} and {*λ_j_*}, respectively; see below). First, {*u_i_*(*t*)} is slowly integrated, ‘informing’ the competition dynamics about the course of execution of a particular mode *j* via the linear differential equation:
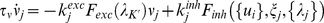
(6)where *τ_ν_* is the time constant and *k_j_^exc^* and *k_j_^inh^* are time scale parameters. *F_inh_*(.) and *F_exc_*(.) are feedback functions, inhibitory and excitatory, respectively. *F_inh_*(.) results in the slow increment of the ‘feedback integrating’ variable *ν_j_* while mode *j* is being executed, whereas *F_exc_*(.) resets *ν_j_* to 0 when the sequence is completed (or equivalently, when the last mode *K′* has been executed). Second, the slow integration above triggers fast transitions of the ‘switching*’* variables {*λ_j_*}:
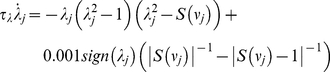
(7)where *τ_λ_* is the time constant, *S*(*ν_j_*) is a sigmoidal function ‘sharpening’ the effect of *ν_j_* and limiting it to the interval [0,1], and *sign*(.) returns the sign of its argument. *λ_j_* transits fast to a point attractor at *λ_j_* = 0 when *S*(*ν_j_*) →1 and, inversely to a point attractor with |*λ_j_*| = 1 when *S*(*ν_j_*) →0. For intermediate values of *S*(*ν_j_*) (far from 0 and 1) the system is bistable; the above transitions are thus characterized by hysteresis. (For more details on the dependence of the phase space structure of equation (7) on parameter *S*(*ν_j_*) as well as the functional forms of the feedback functions *F_inh_*(.), *F_exc_*(.) ,and the sigmoidal *S*(.), see Text S1 as well as Figure S2 of the Supporting Information) Finally, {*λ_j_*} are inserted into the competition equation (5) determining, as mentioned above, the availability of a functional mode to participate in the competition (or inversely its inhibition) via parameters {*L_j_*} as well as the outcome of the competition via parameters {*C_j_*}. The *L*s transit fast between values 0 and 1 following:
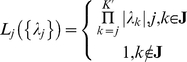
(8)Thus, a functional mode *j* participates in the competition (*L_j_* = 1) when neither *j* nor any of the subsequent modes in the sequence are inhibited. The modes that do not form part of sequence **J** still take part in the competition (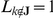) but fail short due to their low {*C_j_*} activations. The {*C_j_*} transit fast to *C_0_* (and vice versa) from a value greater than *C_0_* according to:
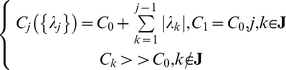
(9)Since *L_j_* = 1 for all modes participating in the competition, the winner *j* at each time moment is the one with 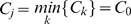.

In sum, the transitions of {*λ_j_*}, {*L_j_*} and {*C_j_*}, occurring due to feedback from the output, affect (the outcome of) the {*ξ_j_*} competition, which in turn selects a different functional mode to dominate the architecture's dynamics at each stage of the sequence. In fact, as the different ingredients of the functional architecture are intricately coupled in various ways, the {*ξ_j_*} competition is effectively influenced by all the relevant variables constituting the architecture. As for the characteristic time scales of each one of these variables and parameters, {*ν_j_*} integrate the output at a time scale much slower than the one of functional modes (*τ_v_*>>*τ_f_*), whereas the transitions of {*λ_j_*}, {*L_j_*} and {*C_j_*}, as well as the evolution of ‘individual competition rounds' are fast (*τ_λ_,τ_c_*<<*τ_f_*). The transition times notwithstanding, during the execution of a particular functional mode the corresponding variables and parameters remain relatively constant. Indeed, the resulting trajectory of the {*ξ_j_*} dynamics, which passes sequentially from the neighborhood of each mode of the sequence (where it dwells for a long time during a mode' activation), exhibits a time structure that is defined across the entire sequence. As such, the effective time scale that determines the serial dynamics (referred to as *τ_ξ_* above) is slow, following approximately the time scale (*τ_ν_*) of the integration in equation (6) (*τ_ξ_*≈*τ_v_*>>*τ_f_*). A glossary of all variables and parameters, as well as the time constants (as used in the simulations) rendering the architecture time scale hierarchical can be found in Table 1.

**Table 1:** Variables, parameters and time scale hierarchy.

	Variables		Parameters	Time constants	Time scale hierarchy
Functional modes	functional dynamics	{*u_i_*}	*µ = *0.1, *µ_e_^lc^* = 0.60, *µ_e_^mn^* = 0.15, *µ_e_^bi^* = 0.45	*τ = *0.1, *τ_f_* = *τ/µ*	*τ_f_* = 1
Operational signals	instant ‘kicks’	{*δ_i_*}	-	*τ_δ_* = 0.1*τ_f_*	*τ_δ_* = 0.1
	WTA competition	{*ξ_j_*}	competition parameters {*L_j_*}, {*C_j_*}	*τ_c_* = 0.1	
Serial dynamics	output integration	{*ν_j_*}	feedback functions *F^j^_inh_*(.), *F_exc_*(.)	*τ_v_* = 10*τ_f_* = 10	*τ_ξ_*≈10 (*τ_ξ_*≈*τ_v_*)
	switching	{*λ_j_*}	sigmoidal function *S*(.)	*τ_λ_* = 0.1	

Table 1 summarizes the state variables and parameters that form the functional architecture as well as the time constants that generate the characteristic time scale hierarchy. All time constants scale with the time constant of the fast contraction onto the manifold *τ*.

### Implementation of cursive handwriting

As proof of concept we demonstrate the application of the functional architecture via a typical example of serial motor behavior, namely cursive handwriting. Here, the state variables are (*x,y,z*), whereas a repertoire of *K* = 37 functional modes is used implementing characters (or parts thereof) modeled as 3-dimensional SFMs. Please note that in choosing *K* = 37, the dynamical repertoire is much larger than the number of modes required to establish the task required (see below), as is typically the case. The manifold, the surface of a cylinder with an ellipsoid basis where dynamics unfolds along the *x*-axis, is chosen to be common for all characters (for implementation reasons but without loss of generality). Thus, the form of the functional dynamics (exemplifying equations (2) and (4)) is: 
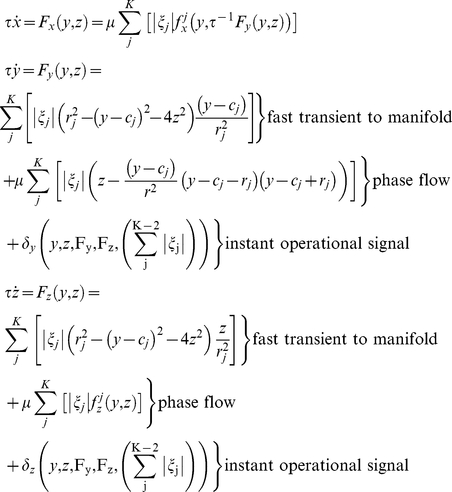
(10)


where *r_i_* is the radius and *c_i_* the center of the manifold. *y* and *z* obey *Excitator*-like dynamics [45] (except for two auxiliary linear point attractor phase flows) that has been proposed as a unifying framework for rhythmic and discrete movements. Depending on whether 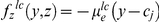, 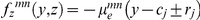, or 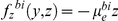, the system exhibits a limit cycle (rhythmic behavior), a point attractor with a separatrix (monostable system with threshold properties), or two point attractors with a separatrix between them (bistable system), respectively. The smallness parameter *µ* provides the time scale separation responsible for the fast contraction on the manifold, while a second parameter *µ_e_^lc/mn/bi^*, guarantees the time scale separation that is necessary for the threshold properties of *Excitator* phase flows. The form of *f_x_^j^*(.) yields the desired letter shapes. The modeling strategy consists in modulating the velocity on the *x*-axis relative to the one on the *y*-axis, according to the direction of velocity and the position of *y*, by means of sigmoidal functions (see Supporting Information Text S1 for detail; Table S1 for parameter values). Finally, the functional mode dynamics (properly scaled and positioned via *r_j_* and *c_j_*, respectively) drives the dynamics on the handwriting workspace (i.e., the *xy*-plane*)*.

**Figure 3:**
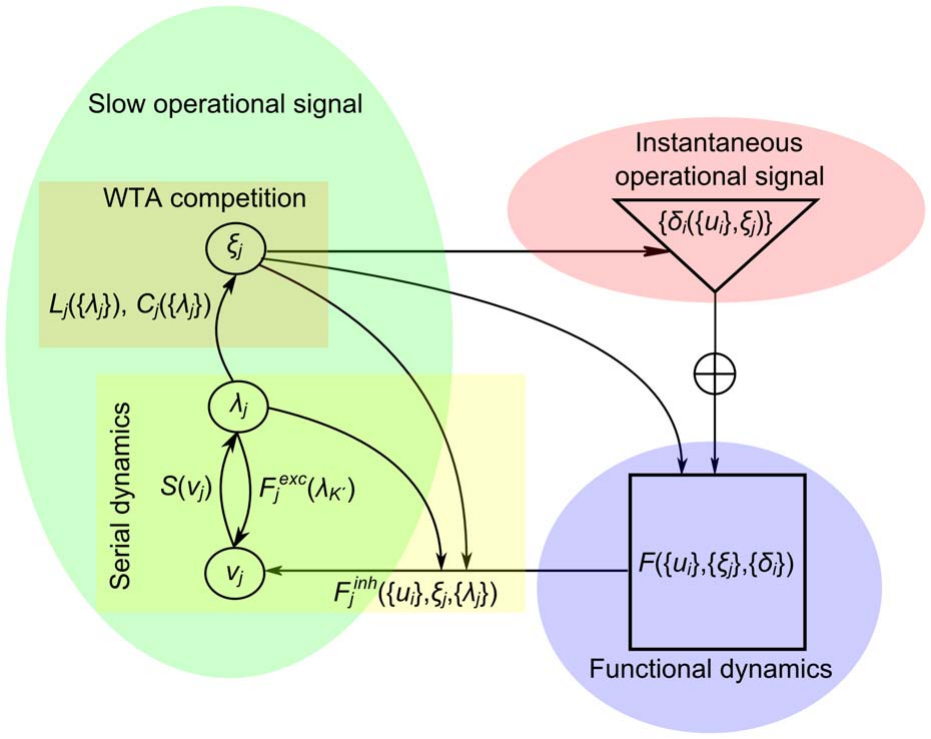
Interactions among the functional architecture's components. The functional dynamics *F*({*u_i_*},{*ξ_j_*},{*δ_i_*}) is fed back to the slow serial dynamics by means of inhibitory feedback, *F_inh_*({*u_i_*},*ξ_j_*,{*λ_j_*}), and is integrated by the *feedback integrating* variable *v_j_*. *v_j_* causes timely fast transitions to the ‘*switching*’ variable *λ_j_* to which it is coupled via the sigmoidal function *S*(*v_j_*) and excitatory feedback *F_exc_*(*λ_K’_*). *λ*s regulate the WTA competition of the {*ξ_j_*} (via *L_j_*({*λ_j_*}) and *C_j_*({*λ_j_*})) that determine which functional mode will dominate the expressed phase flow *F*({*u_i_*},{*ξ_j_*},{*δ_i_*}) at each time moment. The instantaneous operational signal {*δ_i_*({*u_i_*},{*ξ_j_*})} also receives feedback from output {*u_i_*} and is also coupled to the {*ξ_j_*}. Thus, the whole functional architecture becomes an autonomous system. (The index 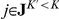 runs among the *K′* modes that participate in a sequence **J** out of a repertoire of *K* modes, and also indicates the specific order of the sequence, i.e., the sequence starts with mode with *j* = 1 and terminates with *j* = *K′*. The index *i* runs among the dimensions of the state variables).

The dynamics of *δ_y,z_* is a function of (*y*,*z*), (*F_y_*, *F_z_*) as well as of{*ξ_j_*}. Its implementation is based on firing a *δ*-‘kick’ when the system approaches a point attractor (see Supporting Information Text S1 for the generation mechanism). Together with *f_ξ_*({*u_i_*}), they render the functional architecture autonomous (Figure 3 illustrates the couplings among the components of the architecture). The joined contributions of dynamics faster (the {*δ*_i_(*t*)} ‘kicks’ and {*ξ_j_*} transitions) and slower (overall {*ξ_i_*(*t*)} dynamics) than the functional modes constitute a time scale hierarchy (see Table 1).

### Simulations and calculation of phase flow variability among trials

The functional architecture is simulated using the Euler-Maruyama method with a fixed time step [79] and normally distributed noise with zero mean and standard deviation *s*. The code is implemented using GNU Scientific Library (GSL) [80] C-code integrated with MATLAB. Noise has an additive contribution to the deterministic dynamics and ensures the robustness of the output by facilitating transitions upon destabilization of a previously stable mode to the next one. Although the effect of noise was not studied systematically, we would like to emphasize that the timing of the transitions depends on feedback rather than on random fluctuations for intermediate amounts of noise, and is thus adequately robust. The parameters were not systematically regulated so as to optimize the output dynamics (a short trial-and-error process was carried out based on visual inspection of the output). The parameters to simulate the word ‘*flow’* presented in the Results were set as follows: noise standard deviation *s* = 0.001, *k_j_^inh^* = [6,12,5,5,2.67,6], and *k_j_^exc^* = [12], [11], [10], [9], [8], [7] for each mode in the sequence, respectively The initial conditions for the functional mode dynamics were *x_0_* = 0, *y_0_* = 0.1, and *z_0_* = −0.1, while those of {*ν_j_*}, {*λ_j_*}, and {*ξ_j_*} where chosen randomly from a uniform distribution in the interval [0,1] for {*ν_j_*} and {*λ_j_*}, and [0,1/*K*] for {*ξ_j_*}.

With regards to the analysis of simulated data, the word ‘*view’* was generated in 100 trials with either the same initial conditions or with initial conditions drawn from a small neighborhood of the phase space with a uniform distribution. All trials where integrated for the same time duration and sampled with the same frequency resulting into data sets with an equal number of data points.

For the analysis, the mean and standard deviation of *y*(*t*), *z*(*t*), *dy*(*t*)/*/dt*, *dz*(*t*)/*/dt* {*ξ_j_*(*t*)}, *δ_y_*(*t*) and *δ_z_*(*t*) were calculated for all trials across each time point, referred to as mean and standard deviation time series, and denoted as *w^µ^*(*t*) and *w^s^*(*t*) respectively (where ‘*w’* may be *y*, *z*, *dy/dt*, *dz/dt ξ_j_*, *δ_y_* or *δ_z_*). (*x* was excluded from this analysis because it does not provide any relevant information about the functional modes phase space geometry since 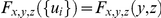 is not a function of *x*). The mean time series was used as a guide in order to estimate the phase space trajectories as well as the flow as follows: For each trial and each time point of the mean time series *y^µ^*(*t*) and *z^µ^*(*t*) (excluding short segments at the beginning and end of the data sets), we searched for the nearest neighbor of (the mean of) *y* and *z* in phase space (i.e., the one with the minimum Euclidian distance) in a time window of *T_w_*  = 300 time points (smaller than half a movement cycle) centered around that time point. Thus, the trials' data sets were rearranged such that their corresponding data points were as close as possible in the *y*-*z* phase plane. Next, the remaining variables of the data sets (*dy/dt*, *dz/dt,*{*ξ_j_*}, *δ_y_* and *δ_z_*) were rearranged accordingly so as to correspond to the related (*y*, *z*) point. We then calculated the mean and standard deviation of the rearranged *y*, *z*, *dy/dt*, *dz/dt*, {*ξ_j_*}, *δ_y_* and *δ_z_* datasets across all trials at each data point, denoted as *w^µ^* and *w^s^* respectively (again, ‘*w’* may be *y*, *z*, *dy/dt*, *dz/dt ξ_j_*, *δ_y_* or *δ_z_*). Notice that *dy/dt* and *dz/dt*, calculated at intermediate steps of the integration algorithm, can only approximate the deterministic phase flow as they contain the additive stochastic contribution of noise to the dynamics, and that the approximation of the integration algorithm is better for choices of smaller time steps *dt*.

## Results

### Simulation of the functional architecture

**Figure 4:**
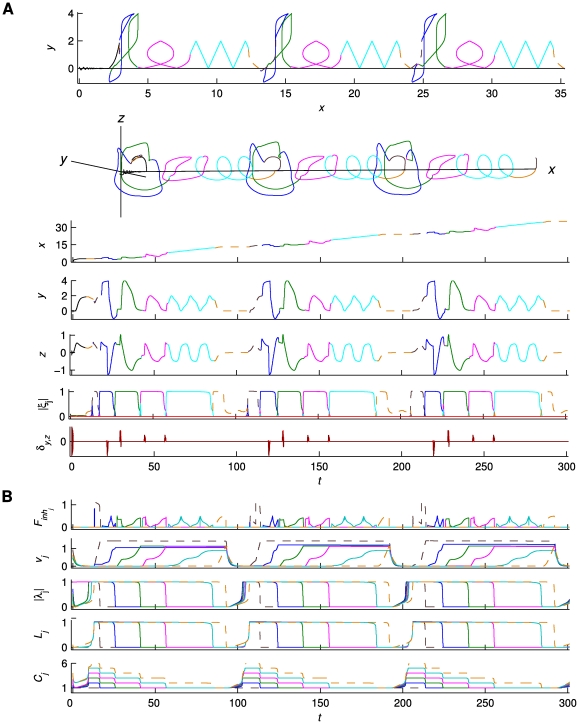
Simulation of the functional architecture generating the word ‘*flow*’. Panel A shows the generation of the word ‘*flow*’ and the operational signals involved. The word is repetitively generated after a short transient (black solid line). Four principle functional modes are used, one for each character (associated with solid blue, green, magenta and cyan lines, respectively), plus two auxiliary ones at the sequence's beginning and end (dotted dark and light brown lines, respectively). From top to bottom: three repetitions of the word in the handwriting workspace (the plane *x*-*y*), the output trajectory in the 3-dimensional functional phase space spanned by state variables *x*, *y* and *z*, followed by their time series, and the time series of the slow (WTA competition coefficients {|*ξ_j_*|}) and the instantaneous (*δ_y,z_* ‘kicks’, light and dark red, respectively) operational signals. The {*ξ_j_*} of the modes that do not participate in the word always have a value close to zero (red line). Panel B shows the feedback loop from the output dynamics to the slow sequential one. From top to bottom: time series of the inhibitory feedback functions *F^j^_inh_*, the slow feedback integrating variables *v_j_*, the absolute values of the (fast) ‘*switching*’ variables *λ_j_*, and the WTA competition parameters *C_j_* and *L_j_*. These quantities vary on the time scale of a whole word (except for *F^j^_inh_* that varies at the time scale of a movement cycle), even if they also contain fast changes during their evolution. The parameter values for this simulation were as follows: noise standard deviation was *s* = 0.001, while *k_j_^inh^*  = [6,12,5,5,2.67,6] and *k_j_^exc^*  = [12], [11], [10], [9], [8], [7] for each mode in the sequence, respectively (only these parameters that have to be manually set prior to a simulation). The initial conditions for the functional mode dynamics were *x_0_* = 0, *y_0_* = 0.1, and *z_0_* = −0.1, while those of {*ν_j_*}, {*λ_j_*} and {*ξ_j_*} where chosen randomly from a uniform distribution in the interval [0,1] for {ν_j_} and {λ_j_}, and [0,1/*K*] for {*ξ_j_*}.

In the following we provide proof of concept that complex functions can be composed of elementary processes organized in an autonomous hierarchy. In the corresponding cursive handwriting illustration, a word is generated via functional modes, each of which ”writes” a character. In other words, functional modes do not code for the individual characters, but rather for the processes involved in generating them. This subtle but fundamental differentiation characterizes our approach towards the emergence of functional dynamics. To be concrete, Figure 4 presents the simulation of the word ‘*flow*’; it shows the output dynamics and operational signals involved (panel A), and the feedback from the output dynamics to the slow sequential {*ξ_j_*} (panel B). Four principal functional modes were used, one for each character: ‘*f’*, ’*l’* and ‘*o’* are implemented using monostable phase flows requiring one *δ-‘*kick’ for the initiation of each movement cycle [45], whereas ‘*w’* is implemented as a limit cycle phase flow (no external timing is required). Two auxiliary (linear fixed point) phase flows are used at the beginning and end of the sequence setting appropriate initial and final conditions. The word is robustly generated repeatedly thrice after a short initial transient due to the random initial conditions.

Notice in particular how Figure 4 illustrates the distinct time scales of the interacting processes: As can be seen in Panel A, the main time scale of the output dynamics pertains to a movement cycle even though a longer (slower) time structure (at the word scale) is present also. This slow time scale dominates the {*ξ_j_*} dynamics. In contrast, *δ_y_*_,*z*_ is much faster than the output. (See Figure S3, Supporting Information for the generation of *δ_y_*_,*z*_). In Panel B, the inhibitory feedback *F_inh_^j^* can be seen to evolve at the (main) time scale of the output, whereas the feedback integrating variables *v_j_*, the absolute values of the ‘*switching’* variables {*λ_j_*} as well as the WTA competition parameters {*C_j_*} and {*L_j_*} are slower (except for fast transitions).

### Class invariance and quantitative variations of functional modes

**Figure 5:**
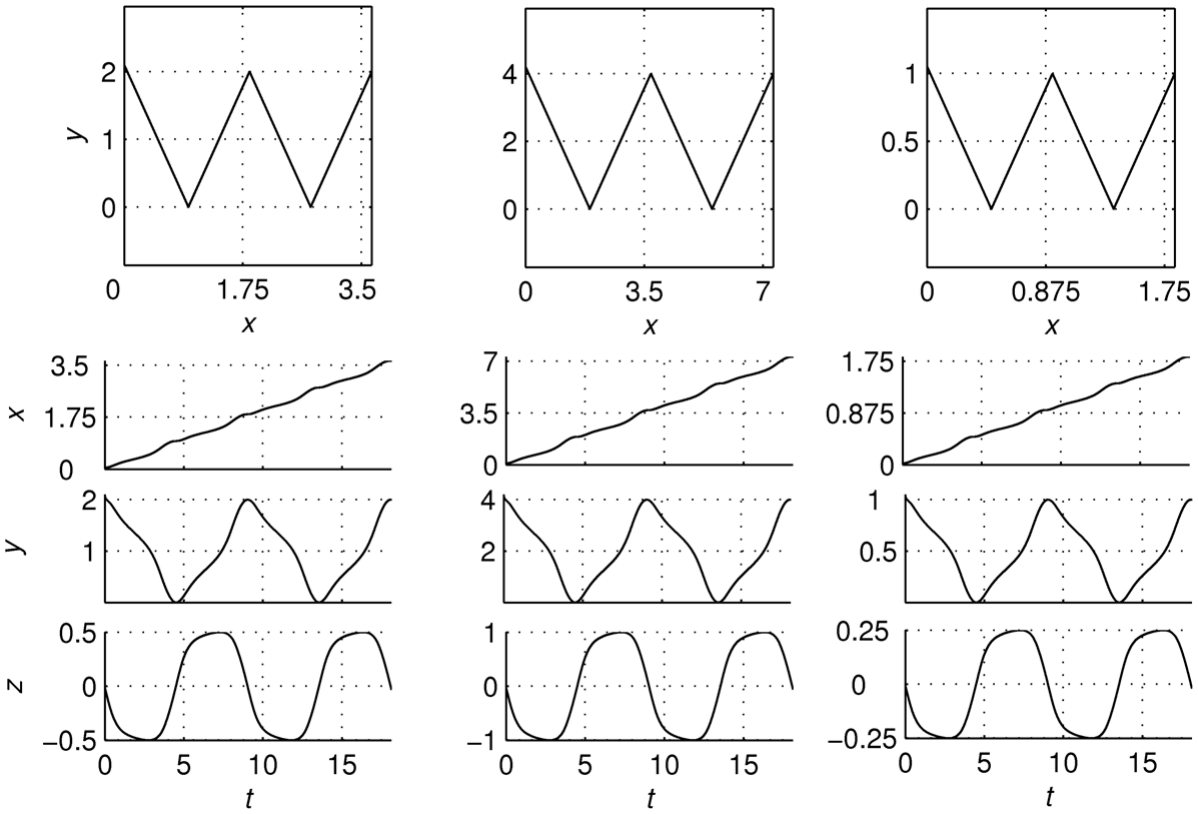
Phase flow scaling. The figure shows the architecture's output generating the character ‘*w*’ with a different movement amplitude at each column: from left to right, the radius of the cylindrical manifold is the default one (*r* = 1), two times larger (*r* = 2) and its half (*r* = 0.5). From top to bottom: the architecture's output in the handwriting workspace (the plane *x*-*y*), and the state variables' time series (*x*, *y*, and *z*). The duration as well as the profile of each stroke's time series is almost identical for all values of the movement amplitude (the isochrony principle).

As mentioned in the Introduction, phase flow topologies provide the means to classify functional modes, and allow for quantitative variation under qualitative invariance. In order to demonstrate this feature, here exemplified by scaling a functional mode’s phase flow with respect to the radius of the manifold (or movement amplitude), we doubled the manifold’s radius of character ‘*w*’ is (default *r* = 1) to *r* = 2, and halved it to *r* = 0.5. All dynamical properties of the output, captured by the shape of its time series, remain invariant (see Figure 5). This scaling of movement velocity with movement amplitude illustrates the so-called isochrony principle [81], which is a well-documented phenomenon in motor behavior and handwriting, in particular.

### Identifying functional modes and operational signals

We next investigate if and how complex processes arising in functional architectures can be decomposed into their dynamical components. Recall, the presence of multiple time scales [65], [66] and nonlinearities render this problem far from trivial. As outlined in the Introduction, this difficulty is evident in the quest for programming units of handwriting, and reflected in the numerous different proposals thereto [67]–[74]. Under the assumption that complex processes are composed of invariant functional modes (except for transitions) and context-specific operational signals, one would expect locally increased variability among trials’ observable trajectories (i.e., pertaining to the functional modes) where operational signals become effective. Moreover, if these operational signals induce transitions between modes and/or introduce meaningful perturbations, these segments of increased variability should generally be of a shorter duration than the characteristic time scale of functional modes. We tested if these two predictions can be used to separate the effects of operational signals on the architecture’s output from the functional mode dynamics, in order to isolate and identify the latter.

Thereto, 100 trials of the word ‘*view*’ were generated. Its dynamics is composed of functional modes based on a monostable phase flow with the point attractor at the position (y^*^,z^*^) = (2,0) (character ‘*v*’), a bistable phase flow with point attractors at positions (y^*^,z^*^) = (0,0) and (y^*^,z^*^) = (2,0) (character ‘*i*’), another monostable flow with the point attractor at position (y^*^,z^*^) = (0,0) (character ‘*e*’), and a limit cycle phase flow (character ‘*w*’) [45]. Regarding transitions in the corresponding phase space structure, it should be noted that no topological changes (locally around the point attractor) are present in the first and second transition (only quantitative variation occurs), while, in contrast, in the third transition a point attractor destabilizes via a Hopf bifurcation [43] giving rise to a limit cycle. Here, we focus on segments of increased variability (among trials) of the operational signals (corresponding to segments of {*ξ_j_*} transitions or of application of *δ*-‘kicks’) and examine their effects on the output variability.

**Figure 6:**
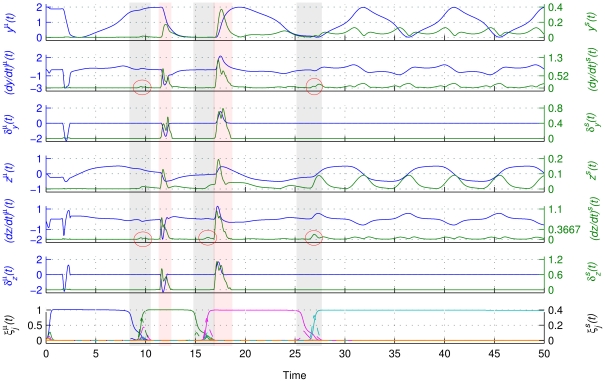
Time series analysis. From top to bottom: means and standard deviations of *y*(*t*) (denoted as *y^µ^*(*t*) and *y^s^*(*t*)) of *dy*(*t*)/*dt* ((*dy*/*dt*)*^µ^*(*t*), and (*dy*/*dt*)*^s^*(*t*)), of *δ_y_*(*t*) (*δ_y_^µ^*(*t*) and *δ_y_^s^*(*t*)), of *z*(*t*) (*z ^µ^*(*t*) and *z^ s^*(*t*)) of *dz*(*t*)/*dt* ((*dz*/*dt*)*^µ^*(*t*), and (*dz*/*dt*)*^s^*(*t*)), of *δ_z_*(*t*) (*δ_z_^µ^*(*t*) and *δ_z_^s^*(*t*)), and of {*ξ_j_*(*t*)} (*ξ_j_^µ^*(*t*) and *ξ_j_^ s^*(*t*)). Means are plotted in blue and standard deviations in green except for the graph of {*ξ_j_*(*t*)} where colors correspond to different modes and where means and standard deviations are plotted with a continuous and dashed line, respectively. Grey and pink shadings focus on the segments of increased {*ξ_j_*(*t*)} and *δ_y_*_,*z*_(*t*) variability, respectively. Notice the strong effect of *δ*-‘kicks’ on the means and standard deviations of the state variables' rates of change ((*dy*/*dt*)*^µ^*(*t*), *dz*/*dt*)*^µ^*(*t*) and (*dy*/*dt*)*^s^*(*t*), (*dz*/*dt*)*^s^*(*t*)). Instead, the variability of {*ξ_j_*(*t*)} (*ξ_j_^s^*(*t*)) has a much weaker effect on (*dy*/*dt*)*^s^*(*t*) and (*dz*/*dt*)*^s^*(*t*), and cannot be unambiguously distinguished from the rest of the (*dy*/*dt*)*^s^*(*t*) and (*dz*/*dt*)*^s^*(*t*) variability.

Figure 6 shows the means and standard deviation time series of all architecture components. It appears that the *δ*-‘kicks’ markedly effect the mean and standard deviation of *dy*(*t*)/*dt* and *dz*(*t*)/*dt* as brief additive contributions. On the contrary, {*ξ_j_*} variability, although affecting the *dy*(*t*)/*dt* and *dz*(*t*)/*dt* standard deviation, this is hardly distinguishable from the standard deviation's variability that is due to the (slightly) different initial conditions and/or stochastic influences.

**Figure 7:**
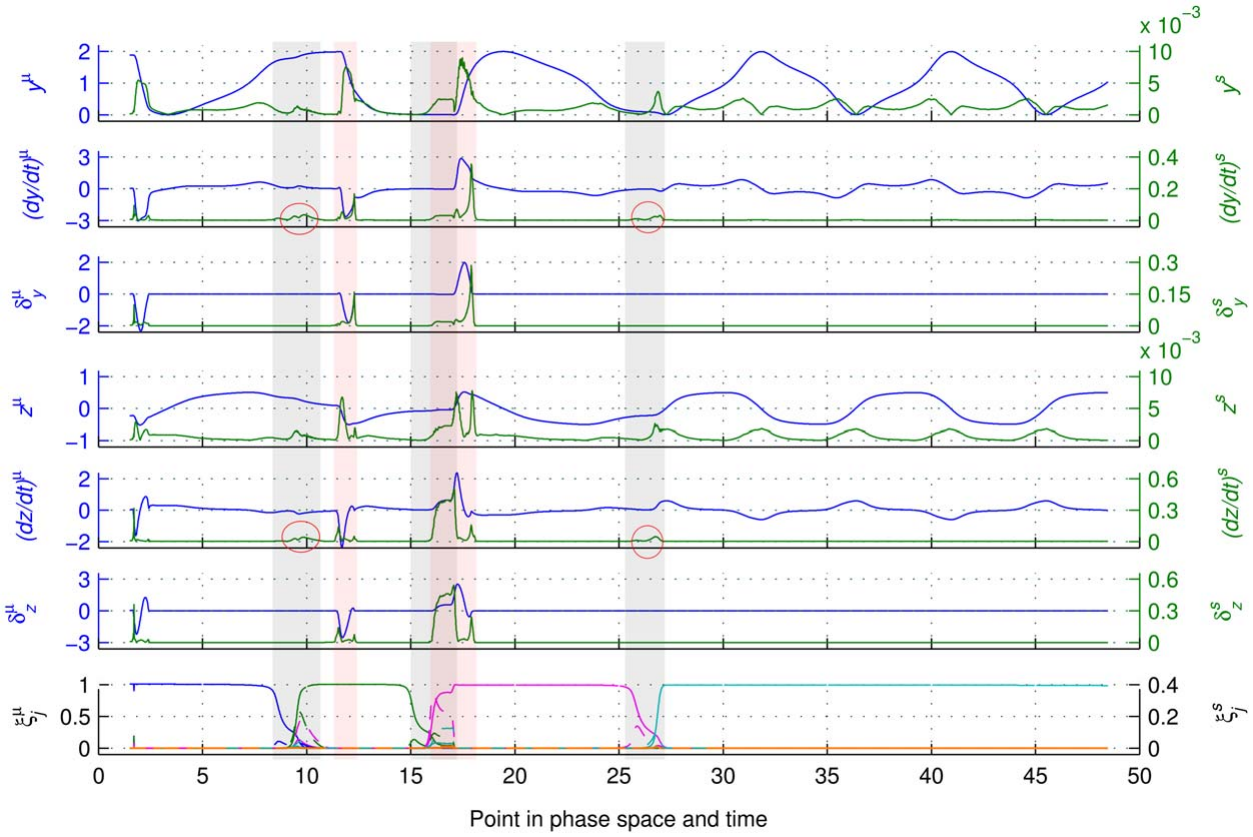
Phase space analysis. From top to bottom: means and standard deviations of *y* (denoted as *y^µ^* and *y^s^*), of *dy*/*dt* ((*dy*/*dt*)*^µ^* and (*dy*/*dt*)*^s^*), of *δ_y_* (*δ_y_^µ^* and *δ_y_^s^*), of *z* (*z ^µ^* and *z^ s^*), of *dz*/*dt* ((*dz*/*dt*)*^µ^* and (*dz*/*dt*)*^s^*), of *δ_z_* (*δ_z_^µ^* and *δ_z_^s^*), and of {*ξ_j_*} (*ξ_j_^µ^* and *ξ_j_^ s^*). Colors, shadings and line styles are similar as in Figure 6. The effect of *δ*-‘kicks’ on the architecture's output is as evident as in Figure 6 (notice also that (*dy*/*dt*)*^s^* and (*dz*/*dt*)*^s^* are almost identical to *δ_y_^s^* and *δ_z_^s^*, respectively, in the segments with a *δ*-‘kick’). The variability of {*ξ_j_*} (*ξ_j_^s^*) that signals mode transitions, now has a significant effect on standard deviations of *dy*/*dt* and *dz*/*dt* that approximate the phase flow. This effect cannot be identified unambiguously in the variability of the trajectory in the phase space (*y^s^* and *z^s^*). At the first transition, the *δ*-‘kick’ variability follows that of the {*ξ_j_*}, and their effects are easily separable. Instead, at the second transition, the mean of the {*ξ_j_*} modulates the standard deviation of *δ_z_* and thereby the one of *dz*/*dt* as well (because of their overlapping in the data set). At the third transition no *δ*-‘kick’ is involved; however, there is still a significant increase in (*dy*/*dt*)*^s^* and (*dz*/*dt*)*^s^* due to the increase in *ξ_j_^ s^*.

Crucially, when performing the same analysis to data sets that have been rearranged so that the corresponding data points refer to neighboring points in phase space within a small time window (see above), the effect of the {*ξ_j_*} becomes evident as well. In addition to the phenomena observable in Figure 6, Figure 7 reveals that an increased {*ξ_j_*} variability (occurring at the moments of transitions between modes) goes hand in hand with an increase in the standard deviation of *dy*/*dt* and *dz*/*dt*. These latter variables (i.e., *dy*/*dt* and *dz*/*dt*) provide an approximation of the phase flow. This phenomenon is caused by the variable changes of the flow due to the {*ξ_j_*} variability ({*ξ_j_*} do not transit identically among trials because of noise) rather than a topological flow change (see Figure S4 of Supporting Information). Note also that the effect of mode transitions cannot be identified unambiguously in the variability of the phase space trajectory (*y^s^* and *z^s^*). (The analysis was performed for noise with standard deviation ten times larger (*s = 0.01*) as well, delivering results that are in general agreement with the above presented ones, as illustrated in Figures S5-7 of the Supporting Information).

In sum, the effects of {*ξ_j_*} transitions or *δ*-‘kicks’ perturbations can be located by focusing on the variability of the output phase flow (approximated here by *dy*/*dt* and *dz*/*dt*) among trials. Both phenomena are short lived due to their intrinsic time scales (fast transients and instantaneous ‘kicks’, respectively) but can be distinguished because the instant *δ*-perturbations are evident in the mean of *dy*/*dt* and *dz*/*dt*, which is not the case for the {*ξ_j_*} transitions. Thus, when mode transitions are identified, a sequence can be segmented into periods where different functional modes dominate the dynamics. Subsequently, evident perturbations can be disregarded as external influences on the functional modes. Finally, the remaining dynamics within a time segment can be considered as an approximately stationary process generated by a particular functional mode. The latter can be recovered by techniques of phase flow reconstruction such as the ones based on Fokker-Planck formalisms [48], [82], [83].

## Discussion

### Functional architectures modeling the phenomenology of human function

We presented a functional architecture comprising multiple subsystems operating on distinct time scales: a dynamical repertoire of functional modes modeled as SFMs, slower operational signals organizing the modes via a Winner-Take-All competition as well as faster ones acting on the modes as meaningful events or perturbations. Crucial to our approach is the idea that functional modes characterize prototypical processes. As proof of concept, we illustrated our approach by generating a cursively written word, a typical instance of serial behavior. Our framework represents a theoretical perspective on process execution and the organization of complex (human) function via a hierarchy of interacting time scales. The approach we adopt, based on modeling motor, perceptual as well as complex cognitive functions in a deterministic, dynamical way, enhances explanatory powers in the context of a specific scientific methodology: abstract dynamics modeling the essentials of biological phenomenology constrain mechanistic models of finer biological detail and suggest possible classes of generating mechanisms; they then feed back to the experimenter with further implications and intuitions based on nonlinear dynamical systems' theory. The presented functional architectures operates on low-dimensional dynamical patterns (functional modes) that explicitly model the specific dynamical structure of an elementary human function (quantitatively as well as qualitatively), and satisfy the requirement for dynamical constituency [44] (viewed here as meaningful structure in the phase (or state) space). By modeling functional modes as dynamical processes instead of states, it may be possible to naturalize (human) function while minimizing complexity reductions typifying traditional approaches [17], [18], [21]. Moreover, the hierarchy of time scales, as a principle of organization, conciliates continuous dynamics with the ‘discrete’ nature of a repertoire of distinct functional modes.

Our approach presents similarities and differences with related ones pushing forward heteroclinic sequences or chaotic attractors to account for brain and cognitive dynamics. For instance, over the last few years, Rabinovich and colleagues developed an approach centering on (cognitive) change via the introduction of heteroclinic cycles [52], [53], which is similar in spirit to ours in several ways, importantly so in focusing on the time structure of cognitive processes. In a nutshell, in their approach the system (cognitive agent) sequentially transits from one unstable equilibrium point (a saddle) to another. Due to the nature of the equilibrium points, the transitions are typically fast and short lived relative to the time spent in their neighborhood (i.e., time-scale separation). A drawback of sequences built on equilibrium points, however, is that their corresponding processes are functionally rather constrained. True, while dynamical objects more complex than (unstable) equilibrium points, such as limit cycles or even chaotic attractors, can be placed at the nodes of a heteroclinic sequence [84], [85], this potential has to our best knowledge not yet been applied to behavioral and cognitive modeling. The limitation to transitions among equilibrium points limits the explicit formulation of the ‘shape’ of given dynamical processes and provides no obvious entry points to their classification. In contrast, our hypothesized range of possible (low-dimensional) dynamical objects (SFM) provides a natural entry point to the classification of cognitive events [84], [85]. Moreover, heteroclinic cycles become slower and slower as a trajectory approaches a saddle point (or subspace), and, importantly, the timing of transitions or the effect of a week perturbation scale with the amount of noise so that robust timing is difficult to achieve. In contrast, in the present implementation, feedback ensures the robust timing of transitions albeit at the expense of an increase in the architecture's dimensionality. The issue of robustness pertains to chaotic attractors (as generated by recurrent neural networks [86], [87]) too: Although they may exhibit dynamics of arbitrary complexity, they are sensitive to initial conditions and thus fail to account for the robustness of human functioning.

Our theoretical framework is complementary to the Bayesian theory proposed in [49]. In a series of papers, hierarchies of transient dynamics were developed in order to account for brain [88], perceptual [50], [51] and behavioral [89] phenomena. In those studies generative models, based on non-linear dynamics, and hierarchical organizations thereof were proposed that can be considered as equivalent to functional modes and architectures respectively. In perception the high levels of the hierarchy encoded slow contextual changes in the environment as the underlying causes of the faster sensory dynamics, the temporal structure of which was captured by the lowest level [50], [51]. In motor behavior slow high-level dynamics were proposed as prior expectations about proprioception, which enslaved the peripheral (faster and low-level) motor system [89] to fulfill them. This Bayesian approach, however, focuses rather on the statistical computation that this dynamics implements in order to ‘tune’ the human and animal brain to the causality structure underlying human–environment interactions (as described by generative models and hierarchies thereof) as well as on the basic principle governing this computation (minimization of uncertainty quantified as free energy [90]). Instead, our work focuses on the actual dynamical objects (and their interactions) that can provide us with dynamical descriptions of human function' phenomenology; in other words, on what we can learn from the proposed generative models. The two approaches, being complementary, could be combined in fruitful ways in future work.

We illustrated our approach by generating an instance of serial behavior, cursive handwriting, as proof of concept. Our model shares common elements with a previously proposed CQ-model for handwriting [64] that reproduced several phenomena observed in the kinematics of human handwriting such as the 2/3 power law as well as the isochrony principle [81]. Attributing different functional roles to the sequence and character generators (serial and functional mode dynamics, respectively) resembles the dual (motor and cognitive) processors model for sequence production [91], [92]). In the latter model, the prime role of the cognitive processor shifts (with practicing) from executing to initiating sequences as the gradual development of motor chunks allows a motor processor to execute them. However, there is no explicit reference to the characteristic time scales of each one of these processes.

The architecture' dynamics (and particularly the functional modes of the handwritten characters) were not constructed with the particular aim to implement biologically realistic kinematics, but to demonstrate the general principles of functional architectures and focus on the interactions between their distinct time scales. However, although biologically realistic dynamics for the modes or a more sophisticated serial dynamics can in principle be constructed based on experimental data, it would not change the nature of our theoretical framework. Moreover, phenomena of dynamics connecting subsequent characters [93], in other words, character variability due to the context of neighboring characters, were not addressed. Doing so either requires a bigger (and more biologically realistic) repertoire of phase flows or the design of a mixed parallel-serial [57], [94] architecture with additional operational signals operating on a time scale similar to the one of the functional modes (*τ_η_*≈*τ_f_*) that would contribute to ‘sewing’ characters into words. However, the inclusion of phase flow modifications at that time scale is the most costly in terms of operational signal complexity [26].

The output {*u_i_*(*t*)} of the architecture represents the observable behavioral trajectory, in our specific implementation, the endpoint trajectory (*x*(*t*),*y*(*t*),*z*(*t*)) of handwriting, whereas the operational signals {*ξ_j_*(*t*)} and {*δ_i_*(*t*)} correspond to internal variables. Only {*u_i_*(*t*)} allows for a direct mapping to behavioral observables, observations of perceptual and other internal contributions must be inferred. The implemented feedback loop that informs the competition process about the evolution of an active functional mode's trajectory likely incorporates both sensory and internal (or ‘planned’) effects; their relative contribution may well depend on the extent to which the movement is automatized. In principle, however, sensory feedback can be explicitly introduced at different levels of the architecture. For instance, it can contribute to the competition between modes, or trigger a fast perturbation to initiate a movement at the correct timing. Sensory feedback can also parameterize the dynamics of a functional mode at time scales similar to the one of functional modes, for instance when a high degree of precision is required.

We further investigated the possibility to identify the functional modes underlying the sequence generation, an endeavor that an experimentalist might find herself faced with. Our results suggest that the various sources contributing to the variance in and across instantiations of a process cannot be unambiguously delineated when focusing on a process' evolution as it unfolds in time. Rather, they urge the experimenter to focus on phase space analysis in order to identify the functional components of serial processes and their interactions. Moreover, our results indicate that unlike phase transitions that occur as the relevant state variable (e.g., relative phase) transits from one stationary value to another, and that are observed in behavioral and sensorimotor coordination [30] and revealed by increased (phase space) trajectory variability, identifying transitions between (possibly non-stationary) processes requires analysis of the variability of the phase flow. To that aim, more elaborate methods of phase flow reconstruction could be considered, such as the one employed in [48], [82], [83] based on Fokker-Planck formulations. However, important modifications or extensions have to be made for those methods to be able to handle non-stationary processes as well [82], [83] and our results may contribute to this end by probing to the importance of time scale separations.

### Neural correlates of functional modes and architectures

The cornerstone of our approach is the SFM concept, according to which the dynamics of a high-dimensional system (such as the embodied brain) temporarily collapses onto a low-dimensional manifold containing a structured functional flow. This vision is in line with reports of network dynamics, dynamical models as well as biological data indicating that the ensemble dynamics of populations of neurons may effectively reduce to a structured flow in phase space (i.e., a functional mode). For instance, recent as well as ongoing work in our lab progresses in designing large scale neural networks of firing rate populations or spiking neurons coding for SFMs and functional architectures [41], [75], [76]. Other (computational) examples in which a network dynamics generates low-dimensional topological objects in phase space are provided in [95]. Real biological networks of spiking neurons have been reported to generate heteroclinic sequences [96]. Also, central pattern generators, i.e., relatively small autonomous neural networks, are typically constrained to produce limit cycle dynamics. An explicit example of the generation of a 3-dimensional closed orbit in phase space generated in a central pattern generator of the lobster stomatogastric ganglion can be found in [96], [97].

Evidence favoring biological realism for the operational signals (slow and fast) can be found in the literature too. In that regard, the time scale hierarchies of the organization of sensorimotor interactions are proposed to be reflected in the hierarchical organization of the nervous system, in particular the cortex [49], [88]. Structurally, the hierarchy is formed via convergence and divergence of forward and backward connections, while their differential functionality introduces a temporal (and spatial) separation of scales of operation. Presumably, (local) processes in the primary areas occur faster than the modulating influences thereon from the higher levels. For instance, oscillation in the human *β* and *γ* band (corresponding roughly to 13–30 Hz and 30–100+ Hz, respectively) are thought to be associated with feature integration (i.e., content related) while the slower *θ* and *α* band (corresponding roughly to 4–8 Hz and 8–13 Hz, respectively) are presumably involved in top-down regulations adjusting the faster processes in a context-dependent fashion [98]. The instantaneous signals *δ*(*t*) have been associated with timing (‘or clock’) mechanisms [48], [99]. In fact, the notion of brief pulses initiating timed movements is well established in the psychological literature [100], [101], and is accompanied by a plentitude of neuro-imaging studies aiming to identify the corresponding anatomical substrate (for a review, see [102]) for which the cerebellum [103]–[105] and basal ganglia [102] have been put forward as candidate structures.

According to the ‘Good Regulator’ theorem (a central theorem in Cybernetics due to Ashby and Conant [106]), any regulator that is maximally successful and simple must be isomorphic with the system being regulated. Whether this applies to the relationship between the neural system (the ‘regulator’ in our case) and human behavior as the SFM framework implies remains an open question. However, initial results of ongoing experimental work on EEG imaging in a behavioral paradigm of rhythmic versus discrete movements [107] and existing literature [37], [38], [108]–[110] are open for interpretation along these lines: In those studies, low-dimensional behavioral patterns and transitions among them (here {*u_i_*} and {*ξ_j_*} dynamics, respectively) were associated with corresponding low-dimensional spatiotemporal modes in EEG and MEG dynamics and their transitions. In particular, preliminary results in [107] reveal that low-dimensional EEG patterns (at the low frequency range) appear to be isomorphic to the behavioral (movement) patterns. How these patterns relate to the oscillations (and synchrony across them) in the *α*, *β*, and *γ*-ranges is still an open question. In any case, the potential isomorphy of dynamics (more specifically phase flows) between brain and behavioral signals offers an intriguing entry point towards the understanding of representation in the human brain.

### Implications for cognitive modeling, learning and engineering

Our framework is compatible with embodied intelligence approaches, since functional modes may be isomorphic to patterns of closed sensorimotor loops or human-environment interactions. The property of dynamical isomorphy or topological equivalence of sensorimotor interactions offers a novel perspective to phenomena such as motor equivalence [8], [111] and sensory substitution [112], [113]. Motor equivalence refers to the fact that humans can accomplish a given goal via different ‘motor means’ as in using different effectors' systems (writing with one's foot), or, in the present context, via different hierarchical organizations [26]. Sensory substitution is the phenomenon that sensorimotor interactions utilizing a given sensory modality can be effectively substituted by other interactions using another modality. According to our approach, what remains invariant among such different behavioral or sensorimotor patterns would be the ‘shape’ of their dynamics, that is, their meaningful structure in the phase space. The argument holds even if one considers the so-called ‘cognitive’ topology [15], [114] to be different than the mathematical one [44], [115].

We demonstrated how the appropriate choice of the functional ‘circuitry’ (i.e., serial dynamics) within the available dynamical repertoire can lead to the emergence of more complex functions such as serial order behavior. The latter is an example of how such ‘circuitries’ among functional modes can prescribe different causal relationships between them, forming a network of elementary processes. Thus, a variety of functional architectures can emerge, even conditional ones, mimicking the IF-THEN rules found in traditional Artificial Intelligence or architectures with coexistence of cooperative and competitive interactions among functional modes. Moreover, although the proposed architecture is presented in a closed form and executes a prescribed serial behavior, internal (e.g., goals, affective inputs) or external (i.e., perceptual) cues could bias the WTA competition via parameters {*L_j_*}, {*C_j_*}, thus (co-)determining the flow within such a network of processes. In that manner, arbitrary decision-making or behavioral sequences (including perception-action coupling) can be modeled, such as stereotypical interactive behaviors (e.g., browsing in the internet or cooking). Additionally, a hierarchy of multiple levels of such functional architectures could be designed in order to account for a repertoire of even more complex functions necessary to model more rich mental/cognitive constructions.

In any case both functional modes and their organizational interactions would result out of a process of pattern formation in structurally coupled agent-environment systems in an autonomous self-organized manner reflecting the agents’ urge to survive or, in other words, conserve its autonomous organization (referred to as autopoiesis [1], [2]). The proposed framework suggests that different processes of adaptation or learning are required for such complex function to emerge. The acquisition of an initial repertoire of elementary functions would precede processes constructing functional architectures allowing for a multitude of complex behaviors. At the same time, the initial repertoire could be extended with new functional modes by composition of existing ones that would either (or not) qualitatively change the constituent modes (see [26]). The latter mechanism could provide us with the means to model phenomena found in cognitive linguistics literature such as conceptual metaphors and blends [14]–[16], [114]. Those are cognitive mechanisms responsible for transferring the causality structure of a conceptual schema (constructed out of generalization over a class of sensorimotor interactions) to another that is defined in a different conceptual space or domain, as well as for the merging of such domains. In this process, the so-called ‘cognitive’ topology is preserved in order to allow for inference in the target domain based on relationships in the source domain. Another interesting question would be whether the learning dynamics themselves could be described by trajectories generated by distinct phase flows in a relevant phase space, corresponding to qualitatively different learning strategies [116].

Functional architectures, besides dealing with some of the most interesting questions in modern science, the ones concerning human function, can also lead to interesting engineering applications in motor or sensory rehabilitation based on motor equivalence and sensory substitution, as well as in Artificial Intelligence and robotics where multi-time scale functional architectures are already being implemented [117],[118]. Combined with their neural network implementations, a novel paradigm of analog biologically inspired computation with possible materializations in integrated circuits, such as Very-Large-Scale Integration (VLSI) [119] ones, may emerge.

## Supporting Information

Figure S1
**Phase space of the WTA system.** Panels A-C show the phase space of a 2-dimensional WTA competition system (*ξ*_1,2_>0) for different values of the ratios *r*_1,2_ = *L*_1,2_/*C*_1,2_ (*L*_1,2_ = 1 always). Red and green curves denote the 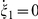 and 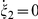 nullclines respectively. On their intersections there is always some equilibrium point. Empty circles denote unstable nodes (this is always the point (0,0)), filled circles point attractors and black rhombs filled with red denote saddle nodes. Arrows describe the vector field whereas blue curves are characteristic trajectories of the evolution of the system (a small asterisk denotes the initial condition). Panel A: *C*_1_ = 1 and *C*_2_ = 2, thus *C*_1_<*C*_2_ and *ξ*_1_ wins the competition. There is a point attractor at the position (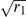,0) = (1,0) (all trajectories converge to it even the ones starting near the *ξ*_2_ node), and a saddle node at (0, 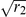) = (0,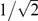). Panel B: *C*_1,2_ = 2. There is no definite winning *ξ_i_*. There is a circle of point attractors because the two circular nullclines are identical. The system can be in any of the states constrained on this circle. A small deviation from this situation will result in the system flowing slowly towards a winning *ξ_i_*. Panel C: *C*_1_ = 2 and *C*_2_ = 1, thus *C*_1_>*C*_2_ and *ξ*_2_ wins the competition. There is a point attractor at the position (0,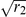) = (0,1) (all trajectories converge to it even the ones starting near the *ξ*_1_ node), and a saddle node at (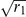,0) = (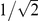,0).(TIFF)

Figure S2 ***λ_j_*** **phase space dependence on parameter** ***S*** **(** ***ν_j_*** **).** Panels from left to right sketch the derivative (red line) of *λ_j_* against itself for values of *S*(*ν_j_*) 0.09, 0.5 and 0.85 respectively. The phase space is 1-dimensional: the *λ_j_* axis. Arrows on this axis describe the vector field. Equilibrium points exist where the derivative curve touches the *dλ_j_/dt* = 0 line. Mutually facing arrows indicate the existence of a point attractor. In the opposite case, there is an unstable equilibrium point. For intermediate values of *S*(*ν_j_*) like *S*(*ν_j_*) = 0.5, there are five equilibrium points: three point attractors at points *λ_j_* = +/−1 and *λ_j_* = 0, and two unstable points separating them. When *S*(*ν_j_*) approaches 0, such as for *S*(*ν_j_*) = 0.09, the previously unstable points disappear and *λ_j_* = 0 destabilizes. Thus, if the system is at that point, it will leave to go to a point where |*λ_j_*| = 1. On the contrary, when *S*(*ν_j_*) approaches 1, such as for *S*(*ν_j_*) = 0.85, it is the *λ_j_* = +/−1 points that destabilize while *λ_j_* = 0 becomes a point attractor (the separating unstable points again disappear). Thus, in this case the system, being in a position where |*λ_j_*| = 1, transits to *λ_j_* = 0. Given that these two *λ_j_* transitions happen for different values of the parameter *S*(*ν_j_*) (due to the bistability for *S*(*ν_j_*) values far from 0 or 1), the system exhibits hysteresis.
(TIFF)

Figure S3 **Generation of the instantaneous operational signal** ***δ_y_***_**,*z***_ **.** From top to bottom panels show *δ*_1_, *δ*_2_ and *δ_cr_* (see equations (C.1,2) of Text S1 of Supporting Information) time series from the simulation of the word ‘*flow’* presented in the main text. Four *δ*-“kicks” are fired for each one of the repetitions of the word. It is *δ_cr_* (that receives input from the other components of the architecture according to (C.2)) the one that triggers pulses for the *δ*_1,2_ excitable system which follows (C.1).
(TIFF)

Figure S4 **Phase space analysis for a non-autonomous slow operational signal and for** ***s*** **= 0.001.** The figure has the same lay out, notation, and color coding as Figure 7 of the main text. {*ξ_j_*(*t*)} are non-autonomous and identical among trials where the mean {*ξ_j_*(*t*)} (*ξ_j_^µ^*) of the autonomous architecture were used The effects of the *δ*-‘kicks’ on the output dynamics are still present. However, since the non-autonomous and identical slow operational signal does not contribute any variability, the effect that had on the variability of the phase flow is attenuated significantly. This is the case even for the third transition, which occurs via a Hopf bifurcation.
(TIFF)

Figure S5 **Time series analysis for** ***s*** **= 0.01**. Figure notation and layout is identical to the ones of Figure 6 of the main text. One can observe the strong effect of *δ*-‘kicks’ to the means and standard deviations of the state variables' rates of change ((*dy*/*dt*)*^µ^*(*t*), *dz*/*dt*)*^µ^*(*t*) and (*dy*/*dt*)*^s^*(*t*), (*dz*/*dt*)*^s^*(*t*)). Instead the variability of {*ξ_j_*(*t*)} (*ξ_j_^s^*(*t*)) has a much weaker effect on (*dy*/*dt*)*^s^*(*t*) and (*dz*/*dt*)*^s^*(*t*) only, which cannot be unambiguously distinguished from the rest of the (*dy*/*dt*)*^s^*(*t*) and (*dz*/*dt*)*^s^*(*t*) variation. Results agree with the ones shown in Figure 5 of the main text.
(TIFF)

Figure S6 **Phase space analysis for** ***s*** **= 0.01**. Figure notation and layout is identical to the ones of Figure 7 of the main text. The effect of *δ*-‘kicks’ on the output of the architecture is as evident as in the simulation of Figure S5 (notice also that (*dy*/*dt*)*^s^* and (*dz*/*dt*)*^s^* are almost identical to *δ_y_^s^* and *δ_z_^s^* respectively at the segments where there is a *δ*-‘kick’). Moreover, the variability of {*ξ_j_*} (*ξ_j_^s^*) that signals mode transitions, has now a significant effect to standard deviations of *dy*/*dt* and *dz*/*dt* that approximate the phase flow. This effect cannot be identified unambiguously in the variability of the trajectory in the phase space (*y^s^* and *z^s^*). At the first transition, the *δ*-‘kick’ variability follows, the {*ξ_j_*} one, and their effects are easily separable. Instead, at the second transition, judging from the shape of the time series, the mean of {*ξ_j_*} modulates the standard deviation of *δ_z_* and through it, the one of *dz*/*dt* as well, because of their overlapping in the data set. Finally, at the third transition, there is no *δ*-‘kick’ involved, however, there is still a significant increase in (*dy*/*dt*)*^s^* and (*dz*/*dt*)*^s^* due to the increase in *ξ_j_^s^*. Results agree with the ones shown in Figure 7 of the main text.
(TIFF)

Figure S7 **Phase space analysis for a non-autonomous slow operational signal and for** ***s*** **= 0.01.** Figure notation and layout is identical to the ones of Figure S4. The effects of the *δ*-‘kicks' on the output dynamics are still present. However, using non autonomous *ξ*s, identical among trials, makes the effect of their variability attenuate significantly (although this attenuation is weaker than the one for *s* = 0.001 shown in Figure S4). This is true even for the third transition where a Hopf bifurcation happens.
(TIFF)

Table S1 **Parameters of functional modes' phase flows.** Table S1 shows the values of the parameters of equations (10) and (D.1,2) of the main text and the Supporting Information Text S1 respectively, which make up the specific phase flows implementing characters' functional modes. Some of the characters require two phase flows in order to be modeled, due to the fact that the dimensionality of the phase flows was constraint to three dimensions, for visualization reasons. Functional modes 36 and 37 just set the system to the position where *z* is 0 and *y* is 0 or 2 respectively (see equations (D.3) of Text S1 of Supporting Information).
(DOC)

Text S1 **Linear stability analysis of the Winner-Take-All competition system and mathematical details for the generation of the sequential dynamics, the** ***δ*** **operational signal and the characters' shapes.**
(DOC)
