# Supplementary figures and images for: Time Scale Hierarchies in the Functional Organization of Complex Behaviors
Source: PLoS Comput Biol. 2011 Sep 29;7(9):e1002198. doi: 10.1371/journal.pcbi.1002198 (PMC3182871; doi:10.1371/journal.pcbi.1002198)

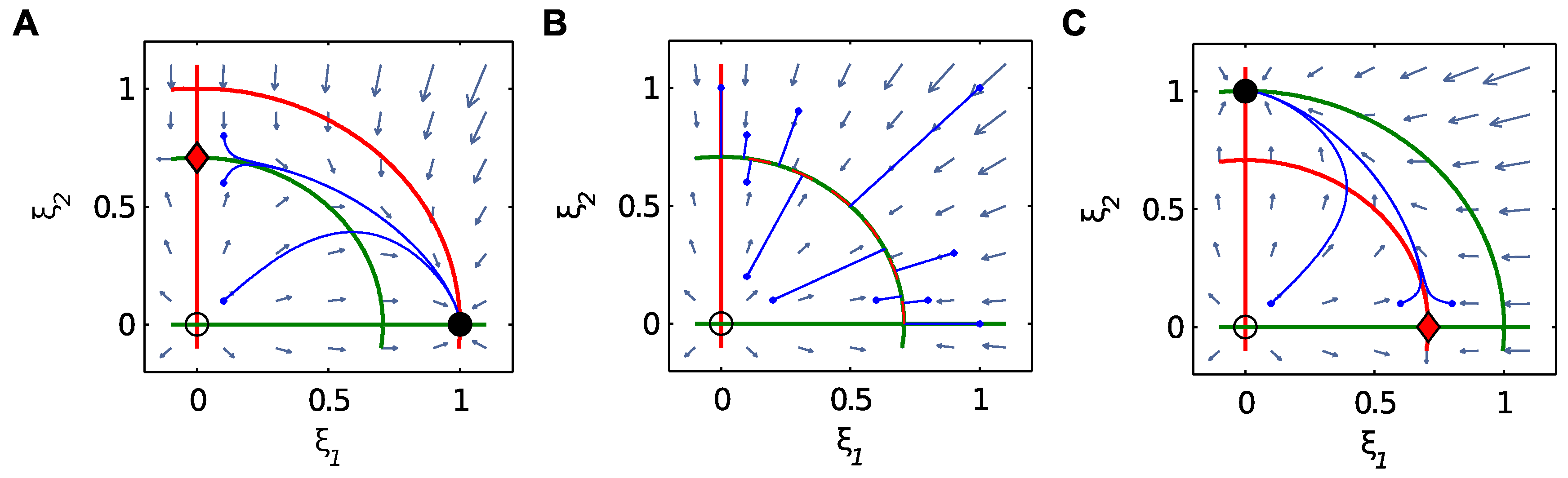

Supplement: Figure S1 — Phase space of the WTA system. Panels A-C show the phase space of a 2-dimensional WTA competition system (ξ 1,2>0) for different values of the ratios r 1,2 = L 1,2/C 1,2 (L 1,2 = 1 always). Red and green curves denote the and nullclines respectively. On their intersections there is always some equilibrium point. Empty circles denote unstable nodes (this is always the point (0,0)), filled circles point attractors and black rhombs filled with red denote saddle nodes. Arrows describe the vector field whereas blue curves are characteristic trajectories of the evolution of the system (a small asterisk denotes the initial condition). Panel A: C 1 = 1 and C 2 = 2, thus C 1<C 2 and ξ 1 wins the competition. There is a point attractor at the position (,0) = (1,0) (all trajectories converge to it even the ones starting near the ξ 2 node), and a saddle node at (0, ) = (0,). Panel B: C 1,2 = 2. There is no definite winning ξi. There is a circle of point attractors because the two circular nullclines are identical. The system can be in any of the states constrained on this circle. A small deviation from this situation will result in the system flowing slowly towards a winning ξi. Panel C: C 1 = 2 and C 2 = 1, thus C 1>C 2 and ξ 2 wins the competition. There is a point attractor at the position (0,) = (0,1) (all trajectories converge to it even the ones starting near the ξ 1 node), and a saddle node at (,0) = (,0). (TIFF) [file pcbi.1002198.s001.tif]

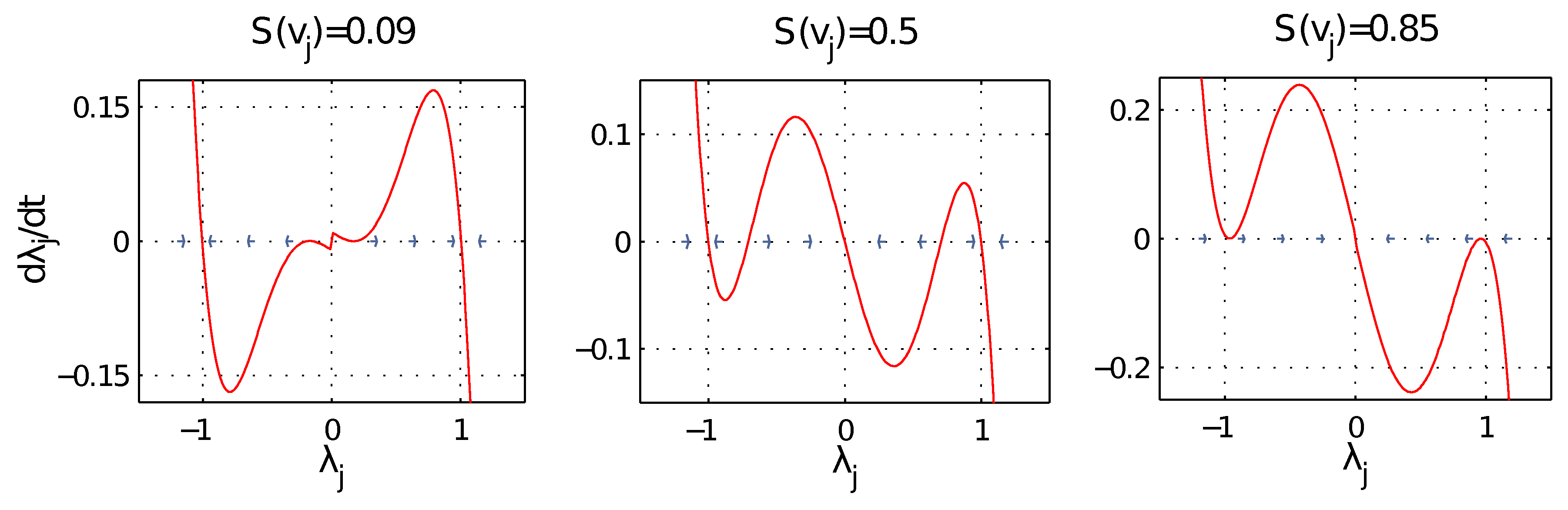

Supplement: Figure S2 — λj phase space dependence on parameter S ( νj ). Panels from left to right sketch the derivative (red line) of λj against itself for values of S(νj) 0.09, 0.5 and 0.85 respectively. The phase space is 1-dimensional: the λj axis. Arrows on this axis describe the vector field. Equilibrium points exist where the derivative curve touches the dλj/dt = 0 line. Mutually facing arrows indicate the existence of a point attractor. In the opposite case, there is an unstable equilibrium point. For intermediate values of S(νj) like S(νj) = 0.5, there are five equilibrium points: three point attractors at points λj = +/−1 and λj = 0, and two unstable points separating them. When S(νj) approaches 0, such as for S(νj) = 0.09, the previously unstable points disappear and λj = 0 destabilizes. Thus, if the system is at that point, it will leave to go to a point where |λj| = 1. On the contrary, when S(νj) approaches 1, such as for S(νj) = 0.85, it is the λj = +/−1 points that destabilize while λj = 0 becomes a point attractor (the separating unstable points again disappear). Thus, in this case the system, being in a position where |λj| = 1, transits to λj = 0. Given that these two λj transitions happen for different values of the parameter S(νj) (due to the bistability for S(νj) values far from 0 or 1), the system exhibits hysteresis. (TIFF) [file pcbi.1002198.s002.tif]

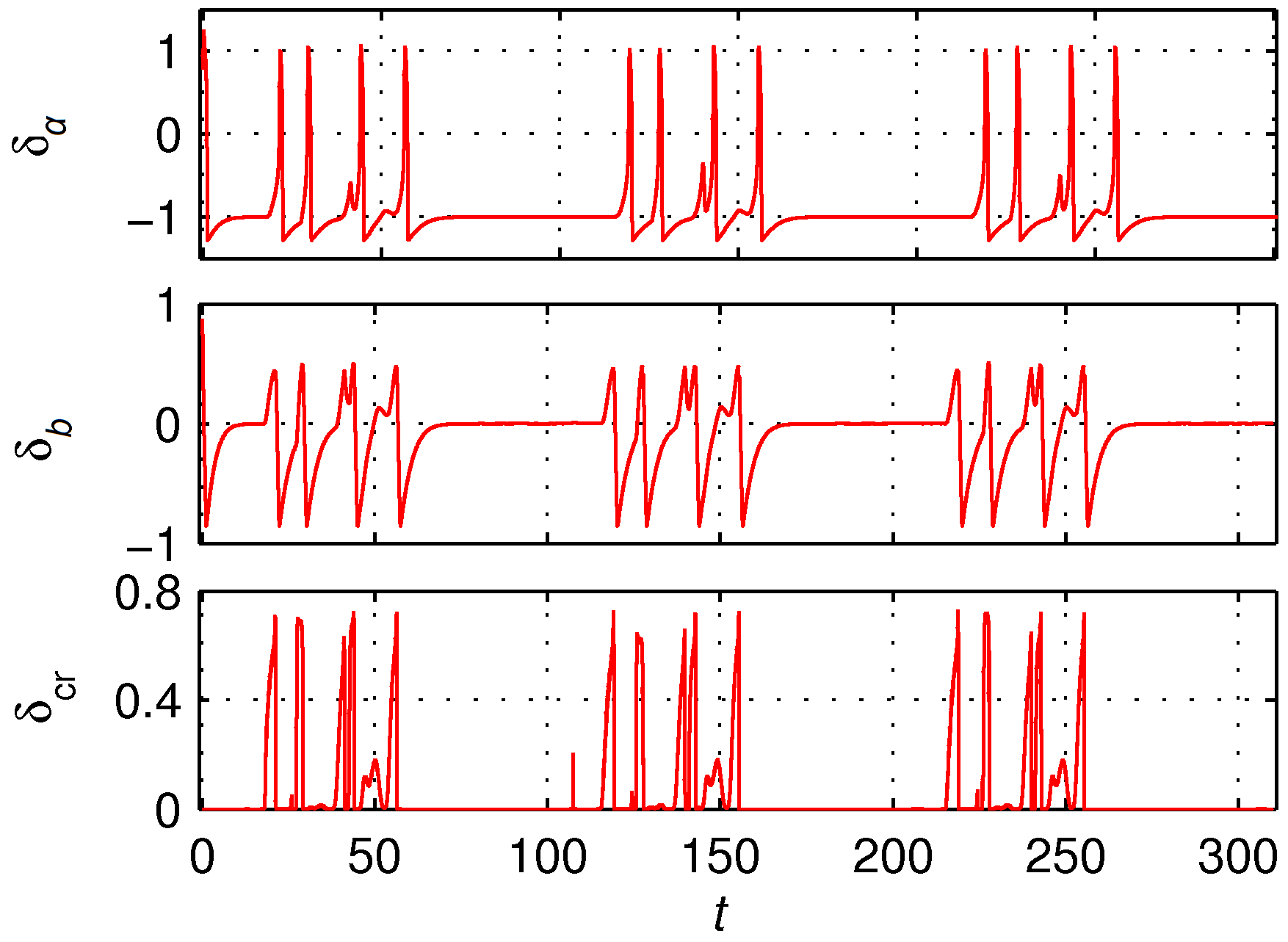

Supplement: Figure S3 — Generation of the instantaneous operational signal δy ,z . From top to bottom panels show δ 1, δ 2 and δcr (see equations (C.1,2) of Text S1 of Supporting Information) time series from the simulation of the word ‘flow’ presented in the main text. Four δ-“kicks” are fired for each one of the repetitions of the word. It is δcr (that receives input from the other components of the architecture according to (C.2)) the one that triggers pulses for the δ 1,2 excitable system which follows (C.1). (TIFF) [file pcbi.1002198.s003.tif]

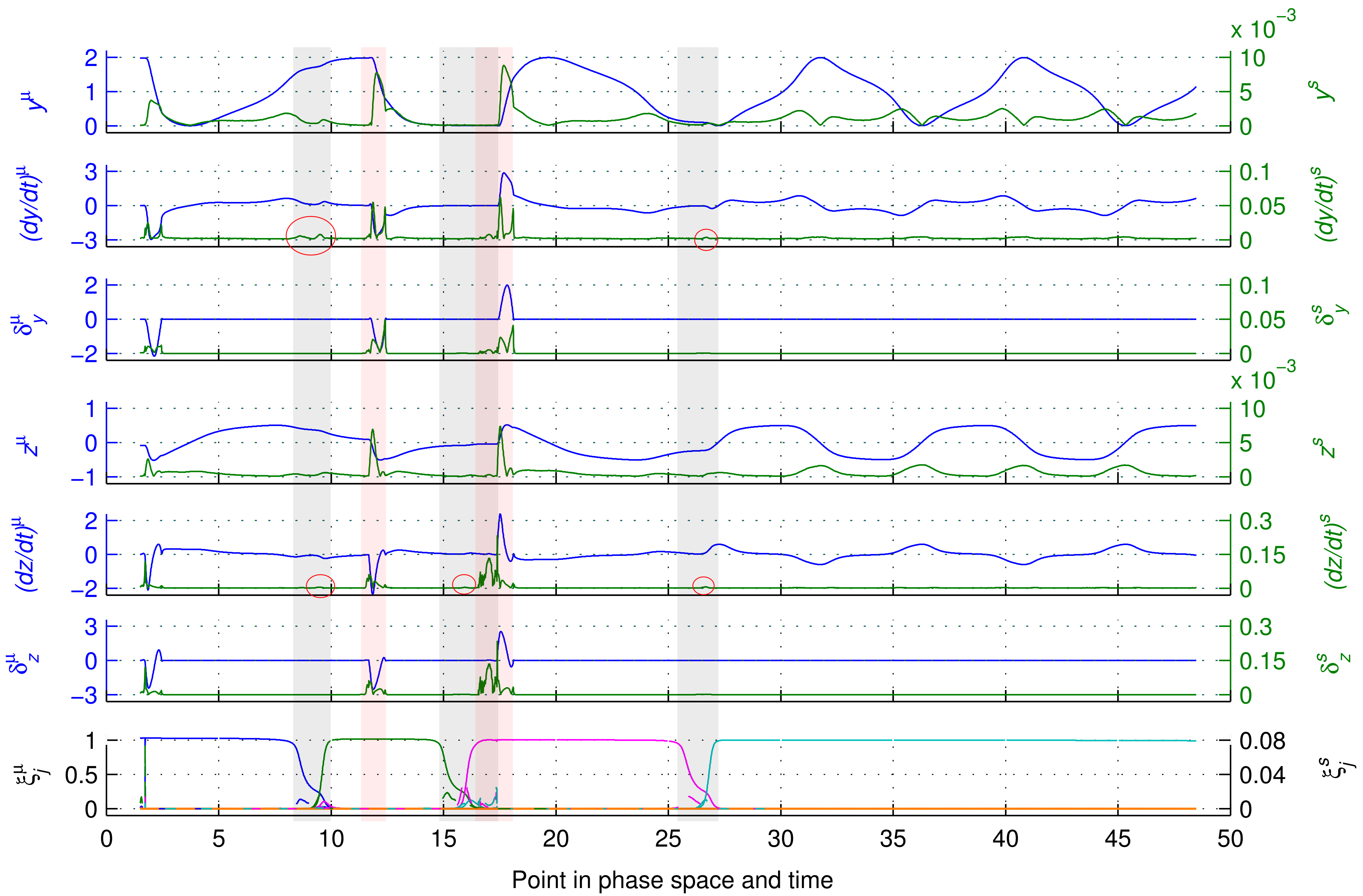

Supplement: Figure S4 — Phase space analysis for a non-autonomous slow operational signal and for s = 0.001. The figure has the same lay out, notation, and color coding as Figure 7 of the main text. {ξj(t)} are non-autonomous and identical among trials where the mean {ξj(t)} (ξjµ) of the autonomous architecture were used The effects of the δ-‘kicks’ on the output dynamics are still present. However, since the non-autonomous and identical slow operational signal does not contribute any variability, the effect that had on the variability of the phase flow is attenuated significantly. This is the case even for the third transition, which occurs via a Hopf bifurcation. (TIFF) [file pcbi.1002198.s004.tif]

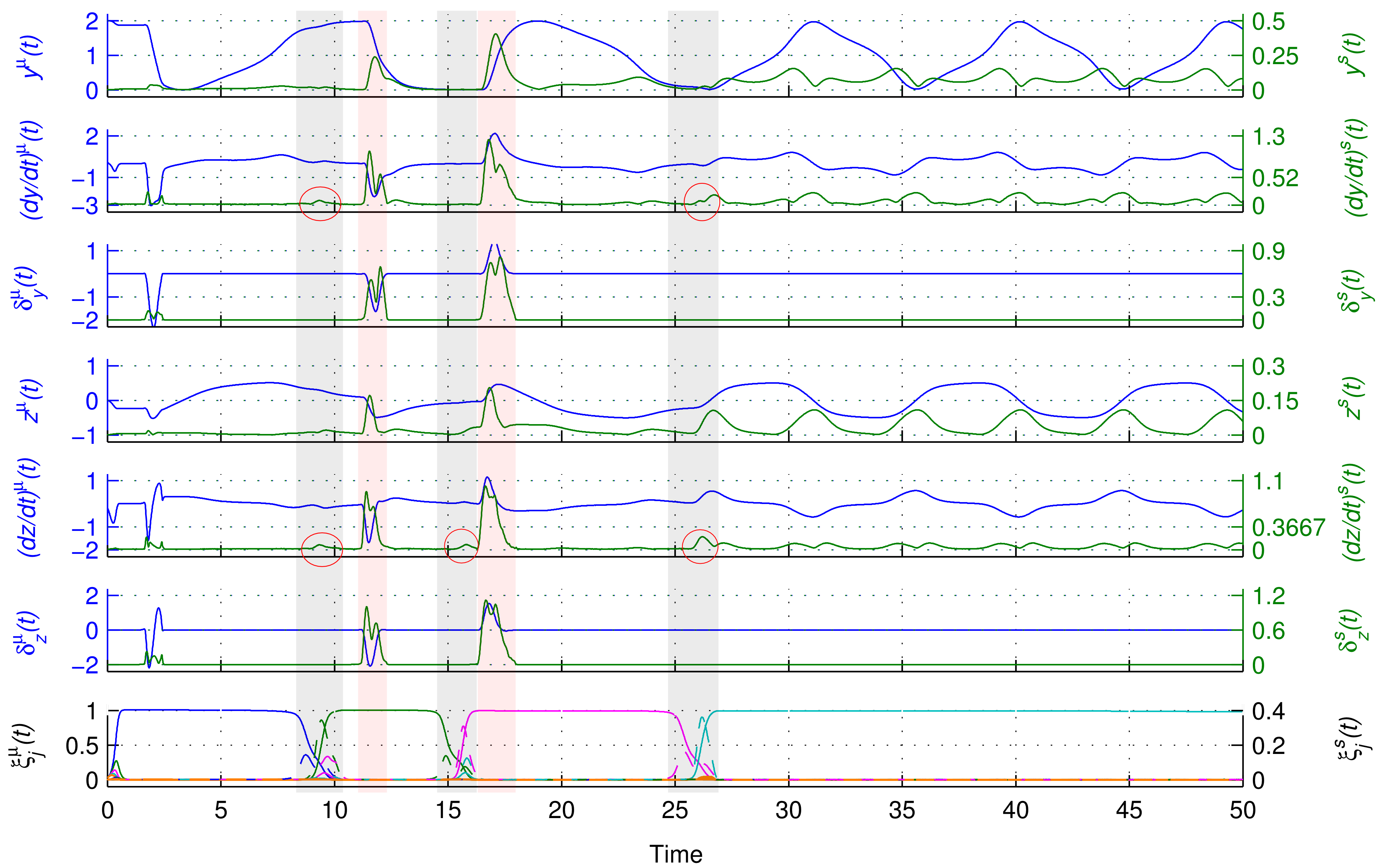

Supplement: Figure S5 — Time series analysis for s = 0.01. Figure notation and layout is identical to the ones of Figure 6 of the main text. One can observe the strong effect of δ-‘kicks’ to the means and standard deviations of the state variables' rates of change ((dy/dt)µ(t), dz/dt)µ(t) and (dy/dt)s(t), (dz/dt)s(t)). Instead the variability of {ξj(t)} (ξjs(t)) has a much weaker effect on (dy/dt)s(t) and (dz/dt)s(t) only, which cannot be unambiguously distinguished from the rest of the (dy/dt)s(t) and (dz/dt)s(t) variation. Results agree with the ones shown in Figure 5 of the main text. (TIFF) [file pcbi.1002198.s005.tif]

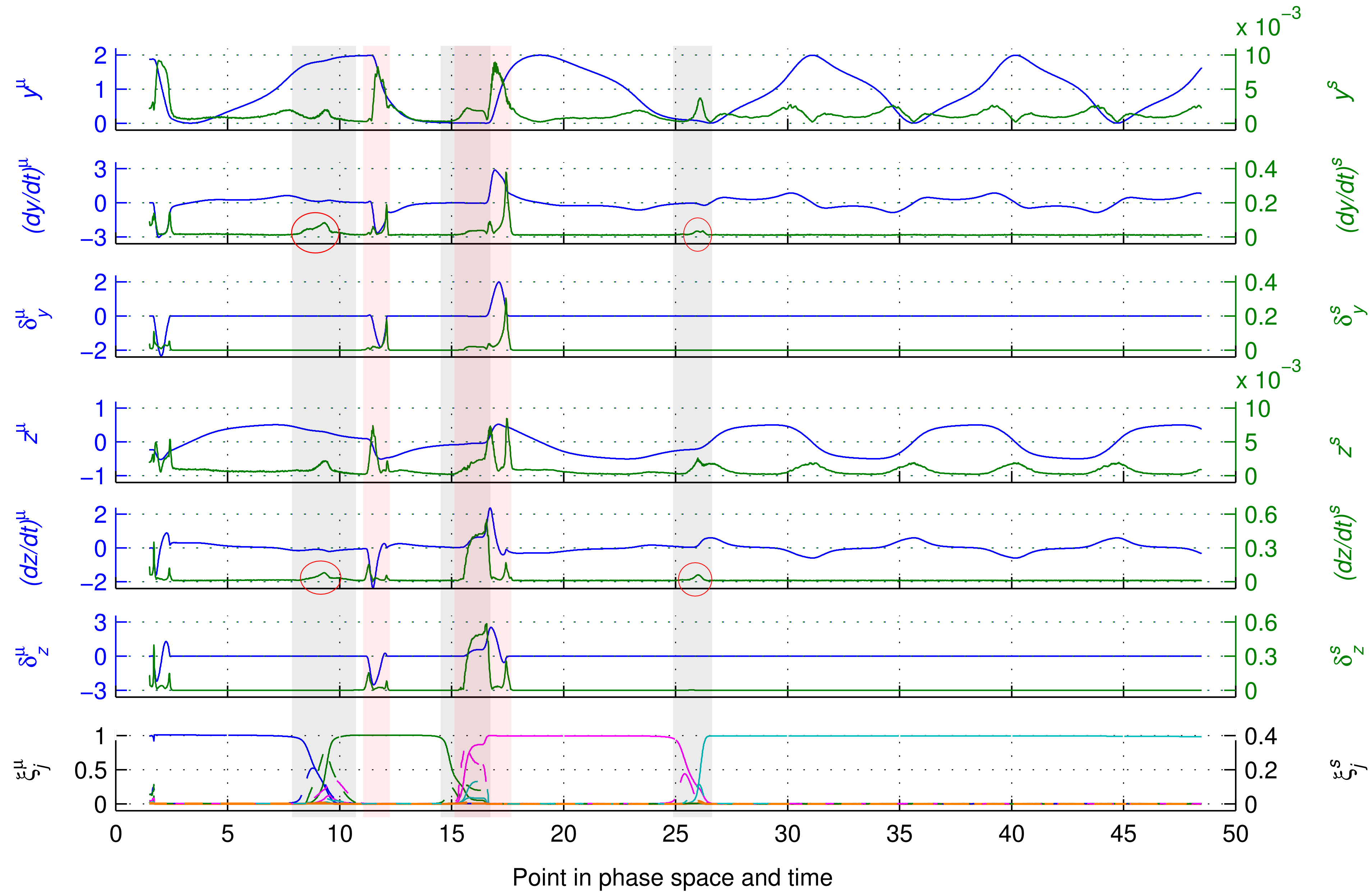

Supplement: Figure S6 — Phase space analysis for s = 0.01. Figure notation and layout is identical to the ones of Figure 7 of the main text. The effect of δ-‘kicks’ on the output of the architecture is as evident as in the simulation of Figure S5 (notice also that (dy/dt)s and (dz/dt)s are almost identical to δys and δzs respectively at the segments where there is a δ-‘kick’). Moreover, the variability of {ξj} (ξjs) that signals mode transitions, has now a significant effect to standard deviations of dy/dt and dz/dt that approximate the phase flow. This effect cannot be identified unambiguously in the variability of the trajectory in the phase space (ys and zs). At the first transition, the δ-‘kick’ variability follows, the {ξj} one, and their effects are easily separable. Instead, at the second transition, judging from the shape of the time series, the mean of {ξj} modulates the standard deviation of δz and through it, the one of dz/dt as well, because of their overlapping in the data set. Finally, at the third transition, there is no δ-‘kick’ involved, however, there is still a significant increase in (dy/dt)s and (dz/dt)s due to the increase in ξjs. Results agree with the ones shown in Figure 7 of the main text. (TIFF) [file pcbi.1002198.s006.tif]

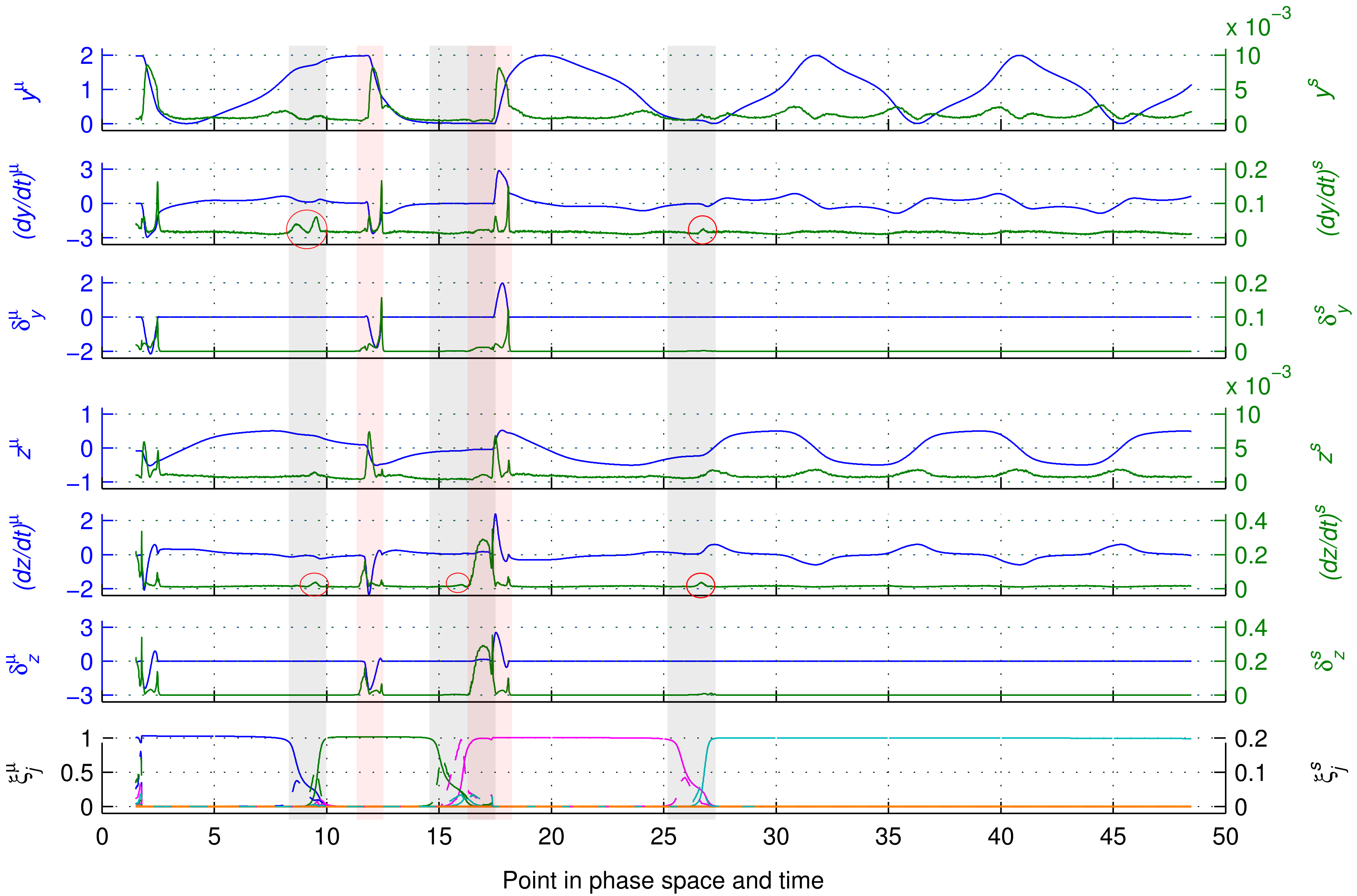

Supplement: Figure S7 — Phase space analysis for a non-autonomous slow operational signal and for s = 0.01. Figure notation and layout is identical to the ones of Figure S4. The effects of the δ-‘kicks' on the output dynamics are still present. However, using non autonomous ξs, identical among trials, makes the effect of their variability attenuate significantly (although this attenuation is weaker than the one for s = 0.001 shown in Figure S4). This is true even for the third transition where a Hopf bifurcation happens. (TIFF) [file pcbi.1002198.s007.tif]
