# Supplementary material for: Time Scale Hierarchies in the Functional Organization of Complex Behaviors
Source: PLoS Comput Biol. 2011 Sep 29;7(9):e1002198. doi: 10.1371/journal.pcbi.1002198 (PMC3182871; doi:10.1371/journal.pcbi.1002198)
Supplement: Table S1 — Parameters of functional modes' phase flows. Table S1 shows the values of the parameters of equations (10) and (D.1,2) of the main text and the Supporting Information Text S1 respectively, which make up the specific phase flows implementing characters' functional modes. Some of the characters require two phase flows in order to be modeled, due to the fact that the dimensionality of the phase flows was constraint to three dimensions, for visualization reasons. Functional modes 36 and 37 just set the system to the position where z is 0 and y is 0 or 2 respectively (see equations (D.3) of Text S1 of Supporting Information). (DOC) [file pcbi.1002198.s008.doc]

**Table S1.** Parameters of functional modes’ phase flows

| ***j*** | **f.m.^1^** | **p.f.^2^** | ***k_xj_*** | ***c_j_*** | ***r_j_*** | ***f_j_*_1_*^p^*** | ***c_j_*_1_*^p^*** | ***t_j_*_1_*^p^*** | ***k_j_*_1_*^p^*** | ***f_j_*_2_*^p^*** | ***c_j_*_2_*^p^*** | ***t_j_*_2_*^p^*** | ***k_j_*_2_*^p^*** | ***f_j_*_2_*^n^*** | ***c_j_*_1_*^n^*** | ***t_j_*_1_*^n^*** | ***k_j_*_1_*^n^*** | ***f_j_*_2_*^n^*** | ***c_j_*_2_*^n^*** | ***t_j_*_2_*^n^*** | ***k_j_*_2_*^n^*** |
| --- | --- | --- | --- | --- | --- | --- | --- | --- | --- | --- | --- | --- | --- | --- | --- | --- | --- | --- | --- | --- | --- |
| 1 | a1 | lc^3^ | 3 | 1 | 1 | 0 | 5 | 1.5 | 5 | 0 | 0 | 1 | 0 | -4.75 | 0 | 0.5 | 5 | 0 | 0 | 1 | 0 |
| 2 | a2 | bi^4^ | 2 | 1 | 1 | -1 | 2 | 0.75 | -1 | 0 | 0 | 1 | 0 | 0 | 10 | 0.1 | -10 | 0 | 0 | 1 | 0 |
| 3 | b1 | mnp^5^ | 2 | 2 | 2 | 0 | 1 | 1.5 | -1 | 0 | 0 | 2 | 0 | 0 | 6 | 0.5 | -10 | 0 | 0 | 2 | 0 |
| 4 | b2 | mnb^6^ | 2 | 1 | 1 | 0 | -4.5 | 1.5 | 10 | 0 | 0 | 1 | 0 | 0 | 7 | 0.5 | -10 | 0 | 0 | 1 | 0 |
| 5 | c | mnb | 2 | 1 | 1 | 0 | 2.5 | 2.1 | -5 | 0 | 0 | 1 | 0 | 0 | -8 | 1.6 | 5 | 0 | 5 | 0.5 | -5 |
| 6 | d1 | mnb | 2 | 2 | 2 | 0 | 15 | 0.1 | -3 | 0 | 0 | 2 | 0 | 0 | -22 | 0.1 | -3 | 0 | 0 | 2 | 0 |
| 7 | d2 | mnb | 2 | 1 | 1 | 0 | 7.5 | 1.75 | 3 | 0 | 0 | 1 | 0 | 0 | 10 | 0.1 | -10 | 0 | 0 | 1 | 0 |
| 8 | e | mnb | 2 | 1 | 1 | -2 | 2 | 1.7 | -5 | 0 | 0 | 1 | 0 | 0 | -5 | 1.6 | 5 | 0 | 5 | 0.5 | -5 |
| 9 | f | mnp | 3 | 2.5 | 1.5 | 0 | 0.6 | 0.875 | 5 | 0 | 0.5 | 2.125 | -5 | 0 | -5 | 3.875 | 5 | -5 | 0 | -0.875 | 5 |
| 10 | g1 | lc | 3 | 1 | 1 | 0 | 5 | 1.5 | 5 | 0 | 0 | 1 | 0 | -4.75 | 0 | 0.5 | 5 | 0 | 0 | 1 | 0 |
| 11 | g2 | mnp | 2 | 2 | 0 | -1 | 2 | -0.5 | 5 | -2 | 2 | 0.5 | -5 | 0 | -17 | -1.8 | -10 | 0 | 0 | 0 | 0 |
| 12 | h1 | bi | 2 | 2 | 2 | -0.5 | 1 | 2.5 | -1 | 0 | 0 | 2 | 0 | 0 | 0 | 2 | 0 | 0 | 0 | 2 | 0 |
| 13 | h2 | mnb | 1.5 | 1 | 1 | 0 | 7.5 | 1.75 | 3 | 0 | 0 | 1 | 0 | 0 | 1 | 1.75 | 10 | 0 | 0 | 1 | 0 |
| 14 | i | bi | 3 | 1 | 1 | -1 | 2 | 0.75 | -1 | 0 | 0 | 1 | 0 | 0 | 10 | 0.1 | -10 | 0 | 0 | 1 | 0 |
| 15 | j | mnp | 2 | 2 | 0 | -1 | 2 | -0.5 | 5 | -2 | 2 | 0.5 | -5 | 0 | -12 | -1.8 | -10 | 0 | 0 | 0 | 0 |
| 16 | k1 | bi | 2 | 2 | 2 | -0.5 | 1 | 2.5 | -1 | 0 | 0 | 2 | 0 | 0 | 0 | 2 | 0 | 0 | 0 | 2 | 0 |
| 17 | k2 | bi | 3 | 1 | 1 | 0 | 1.1 | 1.9 | -5 | 0 | 0 | 1 | 0 | -2.5 | 2.5 | 1 | -5 | 0 | 0 | 1 | 0 |
| 18 | l | mnb | 1 | 2 | 2 | 0 | 3 | 2 | -5 | 0 | 0 | 2 | 0 | 0 | -5 | 3.5 | 5 | 0 | 5 | 0.5 | -5 |
| 19 | m | lc | 1.5 | 1 | 1 | 0 | 7.5 | 1.75 | 3 | 0 | 0 | 1 | 0 | 0 | 1 | 1.75 | 3 | 0 | 0 | 1 | 0 |
| 20 | n | lc | 2 | 1 | 1 | 0 | 7.5 | 1.75 | 3 | 0 | 0 | 1 | 0 | 0 | 1 | 1.75 | 3 | 0 | 0 | 1 | 0 |
| 21 | o | mnb | 8 | 1 | 1 | 0 | -1 | 1.6 | 5 | 0 | 2.25 | 0.4 | -5 | 0 | -1 | 1.6 | 5 | 0 | 2.25 | 0.4 | -5 |
| 22 | p1 | bi | 6 | 1 | 1 | 0 | 2 | 1 | -3 | 0 | -2 | 1 | 3 | 0 | 0 | 1 | 0 | 0 | 0 | 1 | 0 |
| 23 | p2 | mnp | 6 | 2 | 0 | 0 | 1 | -0.5 | 5 | 0 | -1 | 1 | 5 | 0 | 0 | 0 | 0 | 0 | 0 | 0 | 0 |
| 24 | q1 | lc | 3 | 1 | 1 | 0 | 5 | 0.5 | -5 | 0 | 0 | 1 | 0 | 0 | -5 | 1.5 | 5 | 0 | 0 | 1 | 0 |
| 25 | q2 | mnp | 1 | 2 | 0 | 0 | 1 | -0.5 | 5 | 0 | -1 | 1 | 5 | 0 | 0 | 0 | 0 | 0 | 0 | 0 | 0 |
| 26 | r | mnp | 2 | 1 | 1 | 0 | 7.5 | 1.75 | 3 | 0 | 0 | 1 | 0 | 0 | 1 | 1.75 | 10 | 0 | 0 | 1 | 0 |
| 27 | s | mnb | 6 | 1 | 1 | 0 | 0.75 | 1.75 | -5 | 0 | 0 | 1 | 0 | 3 | -3 | 1.5 | 5 | 0 | -4 | 1 | -5 |
| 28 | t | mnb | 7 | 1 | 1 | 0 | 20 | 2 | 10 | 0 | 0 | 1 | 0 | 0 | -4 | 1.75 | 5 | 0 | 4 | 0.25 | -5 |
| 29 | u | lc | 4 | 1 | 1 | 0 | 7.5 | 0.05 | -5 | 0 | 0 | 1 | 0 | 0 | 1 | 0.25 | -10 | 0 | 0 | 1 | 0 |
| 30 | v | mnp | 2 | 1 | 1 | 1 | 0 | 1 | 0 | 0 | 0 | 1 | 0 | 1 | 0 | 1 | 0 | 0 | 0 | 1 | 0 |
| 31 | w | lc | 1.5 | 1 | 1 | 1 | 0 | 1 | 0 | 0 | 0 | 1 | 0 | 1 | 0 | 1 | 0 | 0 | 0 | 1 | 0 |
| 32 | x | mnb | 5 | 1 | 1 | 1.2 | 0 | 1 | 0 | 0 | 0 | 1 | 0 | -8 | 1 | 1.8 | -10 | 0 | 0 | 1 | 0 |
| 33 | y1 | mnp | 4 | 1 | 1 | 0 | 7.5 | 0.05 | -5 | 0 | 0 | 1 | 0 | 0 | 1 | 0.25 | -3 | 0 | 0 | 1 | 0 |
| 34 | y2 | mnp | 2 | 2 | 0 | -1 | 2 | -0.5 | 5 | -2 | 2 | 0.5 | -5 | 0 | -15 | -1.8 | -10 | 0 | 0 | 0 | 0 |
| 35 | z | bi | 2 | 1 | 1 | 0 | 1 | 1 | -5 | 0 | 0 | 1 | 0 | -1 | 42 | 1.9 | 5 | -10 | 7 | 1.01 | -5 |
| 36 | _^8^ | ln^7^ | 5 | 1 | 1 | 0 | 1 | 0.5 | -5 | 0 | 0 | 1 | 0 | 0 | 1 | 0.5 | -5 | 0 | 0 | 1 | 0 |
| 37 | ^9 | ln | 5 | 1 | 1 | 0 | 1 | 0.5 | -5 | 0 | 0 | 1 | 0 | 0 | 1 | 0.5 | -5 | 0 | 0 | 1 | 0 |

^1^functional mode modeled, ^2^phase flow kind, ^3^phase flows based on limit cycle flows ($f_{z}^{lc}\left(y,z\right)=-\mu_{e}^{lc}\left(y-c_{j}\right)$), ^4^phase flows based on *Excitator*-like (see reference [45] in the main text) bistable flows ($f_{z}^{bi}\left(y,z\right)=-\mu_{e}^{bi}z$), ^5^phase flows based on monostable ([40]) flows with the point attractor at the movement’s peak ($f_{z}^{mnp}\left(y,z\right)=-\mu_{e}^{mn}\left(y-c_{j}-r_{j}\right)$), ^6^phase flows based on monostable flows with the point attractor at the movement’s baseline ($f_{z}^{mnb}\left(y,z\right)=-\mu_{e}^{mn}\left(y-c_{j}+r_{j}\right)$), ^7^phase flow based on a linear point attractor flow, ^8^phase flow setting the system’s state at the baseline, ^9^phase flow setting the system’s state at the movement’s peak

Table S1 shows the values of the parameters of equations (10) and (D.1,2) of the main text and the Supporting Information Text S1 respectively, which make up the specific phase flows implementing characters’ functional modes. Some of the characters require two phase flows in order to be modeled due to the fact that the dimensionality of the phase flows was constraint to three dimensions, for visualization reasons. Functional modes 36 and 37 just set the system to the position where *z* is 0 and *y* is 0 or 2 respectively (see equations (D.3) of Text S1 of Supporting Information).
