# Supplementary material for: Time Scale Hierarchies in the Functional Organization of Complex Behaviors
Source: PLoS Comput Biol. 2011 Sep 29;7(9):e1002198. doi: 10.1371/journal.pcbi.1002198 (PMC3182871; doi:10.1371/journal.pcbi.1002198)
Supplement: Text S1 — Linear stability analysis of the Winner-Take-All competition system and mathematical details for the generation of the sequential dynamics, the δ operational signal and the characters' shapes. (DOC) [file pcbi.1002198.s009.doc]

**Supporting Information Text S1**

1. **Linear stability analysis of the Winner-Take-All competition system**

We assume a dynamical repertoire of *K* distinct functional modes. The competition equations:

have the following nullclines (constraining dynamics for $\xi_{i}\geq0$ and parameters for $L_{i}\geq0,C_{i}>0$):

$$C_{i}\left(\frac{L_{i}}{C_{i}}-\sum_{j}^{N} \xi_{j}^{2}\right)\xi_{i}=0\Rightarrow\left\{\begin{matrix} \xi_{i}=0\text{ } \\ \sum_{j}^{K} \xi_{j}^{2}=\frac{L_{i}}{C_{i}}=r>0 \end{matrix}\right.$$

which allow for the general kind of equilibrium points where:

$$\left\{\begin{matrix} \sum_{i\in S} \xi_{i}^{*2}=\frac{L_{s}}{C_{s}}=r_{s}>0 \\ \xi_{i\notin S}^{*}=0\text{ } \end{matrix}\right.$$

or, in other words, some of the *ξ*s lie on a hypersphere of fixed points *S* and the rest are zero. We can assume that there are *P* such sets of equilibrium points and we index them with *p*. We want to find the requirements for such a set to be locally attractive. In other words we require that after a small perturbation in the neighborhood of the hypersphere, the state of the system returns asymptotically on its surface so that is valid.

For this purpose we introduce the variable:

$$r=\sum_{i\in p} \xi_{i}^{2}$$

and we rewrite the equilibrium points of as:

$$\left\{\begin{matrix} r=r_{p}=\sum_{i\in p} \xi_{i}^{*2}>0 \\ \xi_{i\notin p}^{*}=0\text{ } \end{matrix}\right.$$

Then we apply a perturbation study: we assume that when the system is at the point $\mathbf{\xi}^{*}=\left[\xi_{1}^{*},..\xi_{i}^{*},...,\xi_{K}^{*}\right]$ that satisfies a small perturbation takes it to the new point: $\mathbf{\xi}^{'}=\mathbf{\xi}^{*}+\Delta\mathbf{\xi}=\left[\xi_{1}^{*}+\Delta\xi_{1},..\xi_{i}^{*}+\Delta\xi_{i},...,\xi_{K}^{*}+\Delta\xi_{K}\right]$ where it is:

$\left\{\begin{matrix} \xi_{i\in p}^{'}=\xi_{i\in p}^{*}+\Delta\xi_{i\in p}>0\text{ } \\ \xi_{i\notin p}^{'}=\xi_{i\notin p}^{*}+\Delta\xi_{i\notin p}=\Delta\xi_{i\notin p}>0 \end{matrix}\right.$ .

For the rate of change of the perturbations we have:

and by substituting into , $\frac{L_{i\in p}}{C_{i\in p}}=r_{p}$ and $\frac{L_{i\notin p}}{C_{i\notin p}}=r_{i\notin p}$ we get:

where the indexes of the sums have been separated. Then, we also substitute into and we get:

and by substituting into we finally have:

At the same time for the variable *r* and its perturbation we have:

and by substituting first and then into we get:

.

At this point we introduce into we have:

.

But, one can also calculate (using , and ):

$\begin{matrix} \Delta r=r^{'}-r^{*}\Rightarrow\\ \Delta r=\sum_{i\in p} \xi_{i\in p}^{'2}-\sum_{i\in p} \xi_{i\in p}^{*2}\Rightarrow\\ \Delta r=\sum_{i\in p} \left(\xi_{i\in p}^{*}+\Delta\xi_{i\in p}\right)^{2}-\sum_{i\in p} \xi_{i\in p}^{*2}\Rightarrow\\ \Delta r=\sum_{i\in p} \xi_{i\in p}^{*2}+2\sum_{i\in p} \xi_{i\in p}^{*}\Delta\xi_{i\in p}+\sum_{i\in p} \Delta\xi_{i\in p}^{2}-\sum_{i\in p} \xi_{i\in p}^{*2}\Rightarrow\\ \Delta r=2\sum_{i\in p} \xi_{i\in p}^{*}\Delta\xi_{i\in p}+\sum_{i\in p} \Delta\xi_{i\in p}^{2} \end{matrix}$ MERGEFORMAT

which we introduce into and and we summarize the result as in:

.

Finally, we keep only the first order terms of the perturbations since all others are too small:

.

We conclude, that such set of equilibrium points is locally attractive as long as $r_{p}>r_{i\notin p}$ or in other words if $r_{p}=\max_{k} \left\{r_{k}\right\}\Rightarrow\left(\frac{L}{C}\right)_{p}=\max_{k} \left\{\left(\frac{L}{C}\right)_{k}\right\}$. Moreover, it becomes obvious that the origin $\xi_{i=1...K}^{*}=0$ is an unstable node since the second equation of refers to it for *r_p_*=0.

All other equilibrium points are saddles.

In the functional architecture we present in the main text, *L*s transit fast between the values 0 and 1 deciding which modes take part in the competition while *C*s are ordered with a minimum of *C_o_*. Thus, there is always only one point attractor at the position:

$\left\{\begin{matrix} \xi_{i}^{*}=1,\text{ }C_{i}=\min_{j} \left\{C_{j}\right\} \\ \xi_{j\neq i}^{*}=0\text{ } \end{matrix}\right.$ ,

where *ξ_i_* is the winner of the Winner-Take-All competition.

Finally, Figure S1 of the Supporting Information sketches the phase space of a 2-dimensional WTA competition system for different values of the ratios *r_i_*.

1. **Sequential dynamics**

With regards to the sequential dynamics (implementing the feedback loop from the output of the system to the WTA competition), we define$j=1,..,K'$ the index of the subset of $K'$ functional modes that participate in a sequence out of the repertoire of total *K* modes, whereas it also indicates the order of activation. The whole process is described by equations (B.1-6):

First, the output dynamics is slowly integrated:

$\tau_{\nu}\dot{\nu}_{j}=-k_{j}^{exc}F_{exc}\left(\lambda_{K'}\right)\nu_{j}+k_{j}^{inh}F_{inh}\left(y,z,\xi_{j},\left\{\lambda_{j}\right\}\right)$ ,

where:

$\left\{\begin{matrix} \text{ term 1 term 2 term 3} \\ F_{inh}\left(y,z,\xi_{j},\left\{\lambda_{j}\right\}\right)=\frac{\left|\text{atan}\left(\frac{z}{y}\right)\right|}{\pi}\cdot\left(1+e^{-10\left(\sqrt{r^{2}-y^{2}-4z^{2}}-0.5\right)}\right)^{-1}\cdot\left(1+e^{-10\left(\left|\xi_{j}\right|-\left(1+\sum_{m=1}^{K'} \lambda_{m}\right)^{-1/2}\right)}\right)^{-1} \\ F_{exc}\left(\lambda_{K'}\right)=\left(1+e^{10\left(\lambda_{K'}-0.1\right)}\right)^{-1}\text{ } \end{matrix}\right.$ .

Here, *F_inh_*(.) provides the feedback from the output dynamics in the form of half movement cycles (term 1) as long as the system is on the manifold (term 2) and a *ξ_j_* is adequately activated (term 3), while *F_exc_*(.) restores *v_j_* back to zero when the last mode of the sequence has been executed.

Then, *v_j_* triggers fast transitions to the next equation:

,

where:

$\left\{\begin{matrix} S\left(\nu_{j}\right)=\left(1+\mathrm{e}^{-5\left(\nu_{j}-0.5\right)}\right)^{-1} \\ sign\left(\lambda_{j}\right)=\left\{\begin{matrix} +1,\text{ }\lambda_{j}>0 \\ -1,\text{ }\lambda_{j}>0 \\ \text{ }0,\text{ }\lambda_{j}=0 \end{matrix}\right.\text{ } \end{matrix}\right.$ .

Figure S2 in Supporting Information shows the dependence of the system of equation to the parameter *S*(*ν_j_*). When *S*(*ν_j_*) →1 the system has only one point attractor at *λ_j_*=0, which destabilizes for *S*(*ν_j_*) →0 giving way to point attractors at $\lambda_{j}=\pm1$ (in all cases the bifurcations are saddle-node ones). For intermediate values of *S*(*ν_j_*), all the above point attractors are present. Thus, since transitions happen at different values of *S*(*ν_j_*), this system is also characterized by hysteresis.

Finally, transitions in {*λ_j_*} lead to transitions (also fast) to {*L_j_*} and {*C_j_*} that regulate the participation of a mode into the WTA competition and its winner, through:

,

.

Thus, a functional mode *j* participates in the competition (in which case *L_j_*=1) when neither *j* nor any of the subsequent modes in the sequence are inhibited , while {*C_j_*} are ordered in increasing order from the first to the last mode in the sequence . The winner *ξ_j_* value is given by $\xi_{j}=\left(\frac{L}{C}\right)_{j}=1$when *C*_0_=1.

1. **δ operational signal:**

The generation of the instantaneous operational {*δ_i_*} is based on a Excitator-like (see reference [45] in the main text) 2-dimensional differential equation:

.

This equation describes a monostable system with a separatrix. When the parameter *δ_cr_* exceeds the critical value 0.4, a limit cycle is generated through a Hopf bifurcation (see reference [43] in the main text). The critical parameter follows the next equation:

$\begin{matrix} \left.\delta_{cr}\left(y,z,F_{y},F_{z},\{\left|\xi_{J}\right|\}\right)=\text{ }\left(1+e^{-10\left(0.1-\sqrt{F_{y}^{2}+F_{z}^{2}}\right)}\right)^{-1}\text{ }\times\right\}\text{ term 1} \\ \left.\text{ }\left(1+e^{-10\left(\sqrt{r^{2}-y^{2}-4z^{2}}-0.5\right)}\right)^{-1}\text{ }\times\right\}\text{ term 2} \\ \left.\text{ }\sum_{j} \left(1+e^{-10\left(\left|\xi_{j}\right|-\sqrt{\left(1+\sum_{m=1}^{K'} \lambda_{m}\right)^{-1}}\right)}\right)^{-1}\right\}\text{ term 3} \end{matrix}$ .

In other words, a single *δ*-‘kick’ is fired when the system approaches an equilibrium point (term 1) under the conditions that the state of the system is on the prescribed manifold (term 2) and a *ξ_j_* is adequately activated (term 3). After such a *δ*-‘kick’ the systems leaves the neighborhood of the equilibrium point and *δ_cr_* falls back to 0. Finally, the components of {*δ_i_*} are modulated in order that the direction of the kicks is approximately tangent to the surface of the ellipsoid manifold:

$\left\{\begin{matrix} \delta_{y}=\frac{25\delta_{a}}{\left(1+e^{-10\delta_{a}}\right)\sqrt{y^{2}+z^{2}}}z \\ \delta_{z}=-\frac{25\delta_{1}}{\left(1+e^{-10\delta_{a}}\right)\sqrt{y^{2}+z^{2}}}y \end{matrix}\right.$ .

Figure S3 in Supporting Information shows the time series of *δ_α_*, *δ_b_* and *δ_cr_* for the generation of word ‘*flow*’ in the example of the main text.

1. **Generation of the character shapes**

Character shapes are generated through:

$f_{x}^{j}\left(y,\tau^{-1}F_{y}\left(y,z\right)\right)=k_{x}^{j}\left|\tau^{-1}F_{y}\left(y,z\right)\right|\left(\left(1+e^{-5\tau^{-1}F_{y}\left(y,z,t\right)}\right)^{-1}\sum_{k=1,2} S_{jk}^{p}\left(y\right)+\left(1+e^{5\tau^{-1}F_{y}\left(y,z,t\right)}\right)^{-1}\sum_{k=1,2} S_{jk}^{n}\left(y\right)\right)$ .

The modeling strategy consists in modulating the rate of change of *x* (*f_xj_*(.)) relative to the one of *y* ($\left|\tau^{-1}F_{y}\left(y,z\right)\right|$), according to the direction ($\left(1+e^{\pm5\tau^{-1}F_{y}\left(y,z\right)}\right)^{-1}$) and position ($\sum_{k=1,2} S_{jk}^{p/n}\left(y\right)$) of *y*, by means of sigmoidal functions. *k_x_^j^* is a time constant while the sigmoidal functions are given by:

$S_{jk}^{p/n}\left(y\right)=f_{jk}^{p/n}\text{+}\left(c_{jk}^{p/n}-f_{jk}^{p/n}\right)\left(1+e^{-k_{jk}^{p/n}\left(y-t_{jk}^{p/n}\right)}\right)^{-1}$ .

With regards to the last two auxiliary functional modes that set desired initial and/or final conditions at the movement’s baseline or peak, their phase flows are based on linear point attractor dynamics. Thus, the functional forms to be inserted in equation (10) of the main text in the place of the *Excitator*-like phase flows (the cylindrical manifolds remain the same) are:

$\left\{\begin{matrix} f_{y}^{j}\left(y,z\right)=-\left(y-c_{j}\pm r_{j}\right) \\ f_{z}^{j}\left(y,z\right)=-z\text{ } \end{matrix}\right.$ ,

where the plus sign applies for the baseline flow and the minus for the peak one.

1. **Parameters’ values used in the simulations.**

Table S1 in Supporting Information shows the parameters of the specific instantiations of the phase flows making up the functional modes modeling for the characters.
